# Unveiling the Dynamic Pathways of Metal–Organic Framework Crystallization and Nanoparticle Incorporation for Li–S Batteries

**DOI:** 10.1002/advs.202407984

**Published:** 2024-09-24

**Authors:** Xiaohui Song, Rui Huang, Xingyu Zhang, Qiang Chang, Semi Kim, Daeun Jeong, Qian Hou, Juyeong Kim, Edison Huixiang Ang, Xiaowei Su, Xuyong Feng, Hongfa Xiang

**Affiliations:** ^1^ School of Materials Science and Engineering Hefei University of Technology Hefei Anhui 230009 P. R. China; ^2^ Engineering Research Center of High Performance Copper Alloy Materials and Processing Ministry of Education Hefei University of Technology Hefei 230009 P. R. China; ^3^ School of Mathematics Statistics and Mechanics Beijing University of Technology Beijing 100124 P. R. China; ^4^ Department of Chemistry and Research Institute of Natural Sciences Gyeongsang National University Jinju 52828 South Korea; ^5^ Research Institute of Advanced Chemistry Gyeongsang National University Jinju 52828 South Korea; ^6^ Natural Sciences and Science Education National Institute of Education Nanyang Technological University Singapore 637616 Singapore; ^7^ Anhui Honghai New Materials Co., Ltd Anqing Anhui 246100 P. R. China

**Keywords:** 3D tomography, in situ liquid phase TEM, lithium–sulfur battery, MOF, ultrafast high‐temperature sinter

## Abstract

Metal–organic frameworks (MOFs) present diverse building blocks for high‐performance materials across industries, yet their crystallization mechanisms remain incompletely understood due to gaps in nucleation and growth knowledge. In this study, MOF structural evolution is probed using in situ liquid phase transmission electron microscopy (TEM) and cryo‐TEM, unveiling a blend of classical and nonclassical pathways involving liquid–liquid phase separation, particle attachment–coalescence, and surface layer deposition. Additionally, ultrafast high‐temperature sintering (UHS) is employed to dope ultrasmall Cobalt nanoparticles (Co NPs) uniformly within nitrogen‐doped hard carbon nanocages confirmed by 3D electron tomography. Lithium–sulfur battery tests demonstrate the nanocage‐Co NP structure's exceptional capacity and cycling stability, attributed to Co NP catalytic effects due to its small size, uniform dispersion, and nanocage confinement. The findings propose a holistic framework for MOF crystallization understanding and Co NP tunability through ultrafast sintering, promising advancements in materials science and informing future MOF synthesis strategies and applications.

## Graphical abstract

Utilizing in situ liquid phase transmission electron microscopy and cryo‐TEM, classical and nonclassical pathways in MOF structural evolution are uncovered. Ultrafast high‐temperature sintering uniformly dopes ultrasmall cobalt nanoparticles within nitrogen‐doped hard carbon nanocages, confirmed by 3D tomography. Lithium–sulfur battery tests demonstrate exceptional capacity and cycling stability, signifying significant advancements in materials science and MOF synthesis strategies.

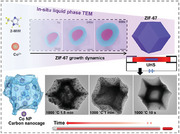

## 1 Introduction

Metal–organic frameworks (MOFs) and covalent organic frameworks (COFs) have surfaced as promising contenders for a diverse range of applications, owing to their exceptional surface area, adjustable pore sizes, and adaptable chemical functionalities.^[^ ^1^, ^2^, ^3^, ^4^, ^5^ ^]^ In recent years, there has been a notable surge in interest in the carbonization of MOFs as a solution to produce carbon‐based materials with customized properties, especially for energy storage applications, notably lithium–sulfur (Li–S) batteries.^[^ ^6^, ^7^, ^8^, ^9^ ^]^ Many efforts have been made to create novel MOF particles with unique morphology based on application requirements. Hence, the understanding of nucleation and growth mechanisms in metal–organic frameworks (MOFs), exemplified by zeolitic imidazolate frameworks (ZIFs), represents a critical frontier in materials science.^[^ ^10^, ^11^ ^]^ Despite significant research efforts and insights gained from in situ liquid phase transmission electron microscopy (TEM) studies,^[^ ^12^, ^13^, ^14^ ^]^ a comprehensive understanding of ZIF nucleation and growth remains elusive. While existing literature offers valuable glimpses into these processes,^[^ ^15^, ^16^, ^17^ ^]^ inconsistencies and gaps persist, hindering the development of precise control strategies for MOF synthesis and application.

One of the primary challenges in elucidating ZIF nucleation and growth mechanisms stems from the inherent complexity of MOF formation.^[^ ^18^, ^19^, ^20^ ^]^ MOFs comprise intricate networks of metal ions or clusters coordinated by organic linkers, forming highly porous structures with tunable properties.^[^ ^2^, ^21^ ^]^ The formation of these frameworks involves a series of dynamic processes, including nucleation, growth, and crystallization, influenced by various factors such as precursor chemistry, solvent environment, and reaction kinetics.^[^ ^22^, ^23^, ^24^ ^]^ While conventional nucleation theories provide a framework for understanding these processes, MOF synthesis often deviates from classical nucleation models due to the multiple competing pathways and intermediate species.^[^ ^25^, ^26^, ^27^ ^]^ As reported, most MOF crystallizations are categorized as non‐classical, as they include intermediates and irregular final crystal structures and morphologies that cannot be explained with classical nucleation theory.^[^ ^19^, ^28^, ^29^ ^]^ To understand the complexity of MOF nucleation and growth, lots of experimental and instrumental methods to obtain a thorough understanding of the mechanisms and kinetics were established. For example, mass spectrometry (MS) provides compositional and structural information of the initial building units in solutions that occur prenucleation.^[^ ^30^, ^31^ ^]^ Such studies enable a molecular understanding of the mechanisms and dynamics in which metastable phases such as particles form. Nuclear magnetic resonance (NMR) is typically used to investigate prenucleation and metastable intermediates, but can also be used to study final MOF crystals and interactions with guest species.^[^ ^32^, ^33^, ^34^ ^]^ Infrared (IR) and Raman spectroscopy can provide information on the molecular bindings occurring in solution and various phases in MOF formation, through measurements of either scattering or absorption of the vibrational modes of the chemical bonds.^[^ ^35^, ^36^, ^37^ ^]^ Scattering techniques (Light scattering, X‐ray scattering, Neutron scattering) can be performed on MOFs to obtain averaged ensemble data of the nucleation and growth kinetics of crystallization, as well as measuring the evolution of particle size, morphology, and distribution.^[^ ^38^, ^39^, ^40^ ^]^ However, each above instrumentation method cannot capture the broad spectrum of the crystallization process from initial building units to final bulk crystal, especially the reported metastable phases.

Additionally, the MOF precursor undergoes heating in a conventional oven at relatively low to moderate temperatures, typically within the range of 200–800 °C, for an extended duration ranging from several hours to days.^[^ ^41^, ^42^, ^43^, ^44^ ^]^ The gradual heating process facilitates the step‐by‐step decomposition and elimination of organic ligands from the MOF framework and leads to the generation of carbon. The MOF‐derived carbons in Li–S batteries provide numerous benefits, such as elevated electrical conductivity, plentiful active sites for sulfur attachment, and improved structural durability throughout the lithiation and delithiation phases.^[^ ^45^, ^46^, ^47^, ^48^, ^49^ ^]^ However, challenges persist with the conventional oven heating method. For example, Co particles form after heating on the surface of MOF particles, whose size and dispersion pattern are difficult to control.

In this work, we employed in situ liquid‐phase transmission electron microscopy (TEM) and cryo‐TEM to investigate the growth dynamics of ZIF‐67, providing new insights into its structural evolution. While numerous studies have explored the growth mechanisms of metal–organic frameworks (MOFs) using similar techniques, our work contributes distinct findings by revealing a combination of classical and nonclassical growth pathways. Specifically, we observed liquid–liquid phase separation, particle attachment–coalescence, and surface layer deposition phenomena that are not always fully addressed in the literature. Subsequently, ZIF‐67 derivatives are produced using the UHS method. Characterizations, including TEM 3D tomography, X‐ray diffraction (XRD), and X‐ray photoelectron spectroscopy (XPS), confirm the formation of hollow porous nanocage, with ultrasmall Cobalt nanoparticles (Co NPs) embedded in nitrogen‐doped carbon matrix uniformly. Li–S battery tests demonstrate excellent electrochemical performance, with discharge‐specific capacities of 785 mAh g^−1^ after 200 cycles at a discharge current of 0.5C and 718 mAh g^−1^ after 200 cycles at a discharge current of 1.0C, whose superior electrochemical performance comes from the catalytic effect of the uniformly distributed Co nanoparticles, and the confinement effect of the nanocage on polysulfides. Our findings highlight the complex interplay between these mechanisms during the growth of ZIF‐67, offering a more nuanced understanding of MOF formation dynamics. Unlike previous studies that may focus on individual aspects of MOF growth,^[^ ^50^, ^51^ ^]^ our research integrates these pathways into a comprehensive model, providing a clearer picture of the evolution of ZIF‐67 structures. This approach not only bridges gaps in current knowledge but also extends the applicability of in situ TEM techniques in studying MOF formation processes, as well as the design of energy storage materials based on MOF derivatives.

## 2 Results and Discussion

### 2.1 ZIF‐67 Nanoparticles Growth Dynamics Under Liquid‐Phase TEM

**Figure 1:**
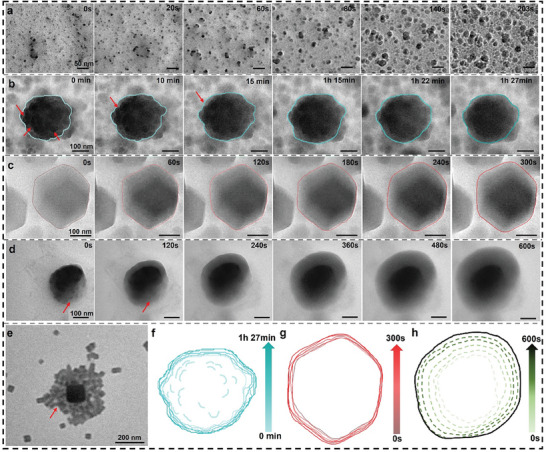
Liquid‐phase TEM imaging and analysis of directional growth of single ZIF‐67 nanoparticles in colloids. a–d) Time‐lapse liquid‐phase TEM images capturing the morphology evolution process of different ZIF‐67 rhombic dodecahedral particles under in situ liquid‐phase TEM. e) cryo‐TEM image showing the attachment of particles on ZIF‐67 cubic to trap the intermediate after 2 min growth (ex situ experiment). f–h) Contour evolution diagrams corresponding to (b–d).

Initially, the pre‐prepared zinc acetate dihydrate solution was rapidly mixed with 2‐methylimidazole and subsequently loaded into a graphene sample holder for observation under transmission electron microscopy. ZIF‐67 exhibits a porous structure resulting from the coordination of cobalt salts with imidazole bonds. From a molecular dynamic viewpoint, the growth of ZIF‐67 can be categorized into nucleation and growth stages. During the nucleation stage, depicted in **Figure** **1**a, numerous small particles emerge and progressively enlarge (Figure S1 and Movie S1, Supporting Information), aligning with classical nucleation theory.^[^ ^52^, ^53^, ^54^ ^]^ Following this, ZIF‐67 progresses into the growth stage, illustrated in Figure 1b, where numerous irregular spheres coalesce to produce a large particle with a rough surface that gradually fuses. The outlines of the particles (highlighted by red arrows) in Figure 1b gradually diminish, ultimately forming a large particle with a smooth surface (Figure S2 and Movie S2, Supporting Information). Figure 1f additionally demonstrates the relative trajectory of the particle morphology change. In Figure 1c, a continuously growing hexagonal particle is observed, developing into an external shell structure. Through quantitative calculations utilizing deep learning techniques,^[^ ^55^, ^56^, ^57^ ^]^ the change in the area of this hexagonal particle can be measured (Figure S3 and Movie S3, Supporting Information). Concurrently, Figure 1g, illustrates the continuous growth process of this particle from a small one to gradually forming a polyhedron. In Figure 1d and its contour map in Figure 1h, numerous small particles are observed, clustering near a large particle (Figure S4 and Movie S4, Supporting Information), akin to the scenario depicted in Figure 1b. Subsequently, these small particles merge to form a shell with a lower contrast. These findings suggest that the growth process of ZIF‐67 encompasses two distinct pathways: one aligns with previous reports, involving nucleation, crystallization, and growth, while the other involves nucleation, attachment, and growth.^[^ ^11^, ^50^ ^]^ Both pathways culminate in rhombic dodecahedral‐shaped nanoparticles, albeit with different sizes. Typically, as depicted in Figure 1e, the cryo‐TEM image shows the intermediates of the nanoparticle attachment (Figure S5, Supporting Information). Typically, cryo‐transmission electron microscopy (cryo‐TEM) was employed to investigate the growth dynamics of ZIF‐67 (zinc‐based zeolitic imidazolate framework). This advanced imaging technique allowed us to visualize the formation and evolution of ZIF‐67 in unprecedented detail under near‐native conditions.^[^ ^58^, ^59^, ^60^ ^]^ Notably, our observations revealed a distinct attachment mode during the growth process: small nanoparticles were found to aggregate and merge into larger cluster intermediates. This intermediate state is characterized by the coalescence of multiple nanoparticles, forming a more substantial cluster that eventually evolves into the final ZIF‐67 structures. This intermediate attachment mode highlights a critical step in the nucleation and growth mechanism of ZIF‐67, providing valuable insights into its formation kinetics and structural development.

Additionally, we performed in situ particle size measurements using NanoZS90, finding that the particles undergo rapid growth within a short timeframe (Figure S6, Supporting Information), indicating that the initial stage nucleation and growth of ZIF‐67 constitute a rapid kinetic process that partially mitigates the effect of the electron beam. To ensure experimental reproducibility and explore sample variations, we conducted multiple experiments. From these trials, we found that the reproducibility rate was ≈60% (6 out of 10).

**Figure 2:**
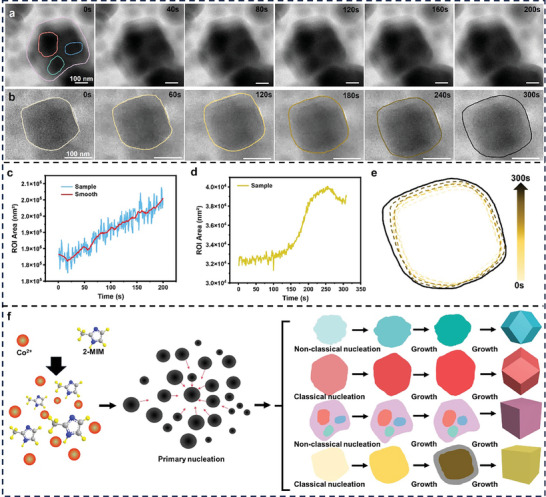
Liquid‐phase TEM imaging of directional growth of ZIF‐67 nanoparticles in colloids via attachment pathway and the ZIF‐67 nanoparticle growth mechanism. a,b) Time‐lapse liquid‐phase TEM images capturing the morphology evolution process of different ZIF‐67 cubic nanoparticles under in situ liquid‐phase TEM. c and d) Graph showing the change in particle area of the ZIF‐67 nanoparticles labeled in Figures a,b over time. e) Schematic diagram showing the contour changes of sample in b. f) Schematic summarizing several different growth modes of ZIF‐67 nanoparticles.

Parallelly, as shown in **Figure** **2**a, we observed three distinct particles gradually converging within a low‐contrast outer shell, with their outer contours gradually changing into indistinct. Furthermore, by analyzing the area change image processed by the U‐net neural network, we quantitatively captured the growth trend of these particles (Figure S7 and Movie S5, Supporting Information). By fitting the area change curve (Figure 2c), we noted a steady increase in the overall particle area, indicating its enlargement and transformation from irregular to cubic morphology, consistent with the attachment growth mode previously identified. Figure 2b illustrates a square particle undergoing continuous enlargement, with a low‐contrast outer shell forming on its outer layer (Figure S8 and Movie S6, Supporting Information). From the area change curve in Figure 2d, we observed uneven growth rates, initially slow growth followed by rapid enlargement, and finally stabilization. In addition, the morphology transitioned from irregular to regular cubic, as depicted in Figure 2e. Figure 2f summarizes the growth modes of all ZIF‐67 nanoparticles based on the findings, illustrating two growth pathways: nucleation‐growth of individual particles and attachment growth of multiple particles, highlighting the diversity in ZIF‐67 growth modes (Supplementary Note 2). Notably, all the above observations shown in Figures 1 and 2 were captured in the same sample area, showing the diversity of MOF particle growth in one batch.

The occurrence of different growth modes in the same liquid phase solution requires investigation into contributing factors. Notably, the attachment growth mode is more prevalent in the center of the electron beam, with stronger radiation, contrasting with areas exposed to weaker electron beam radiation. This phenomenon is likely due to changes in the surface energy of nucleating particles induced by electron beam irradiation, making them more prone to aggregation. To support this explanation, non‐in situ synthesis experiments were conducted. First, product morphology assessments were performed at various time intervals in the ZIF‐67 mother liquor (Figure S9, Supporting Information). TEM images revealed adherence to classical nucleation theory: nucleation commenced with cubic growth, ultimately leading to a dodecahedral structure, with the initial crystals displaying an amorphous structure revealed by SAED and XRD characterizations (Figures S10 and S11, Supporting Information). Then, samples from the ZIF mother liquor at 3 min were analyzed using cryo‐EM techniques, revealing distinct attachment growth behavior (Figure S5, Supporting Information), indicative of a non‐classical nucleation growth mode. This observation aligns with conclusions drawn from in situ liquid‐phase TEM experiments (Supplementary Note 1). Thus, minimizing the occurrence of non‐classical nucleation during ZIF‐67 nanoparticle synthesis is proposed to be advantageous for achieving size control. The findings of this study establish an experimental foundation for future research into morphology control and mechanism elucidation of ZIF‐67 nanoparticles.

### 2.2 Ultrafast Sintering of ZIF‐67 to Tune Co Nanoparticle Size and Dispersion

**Figure 3:**
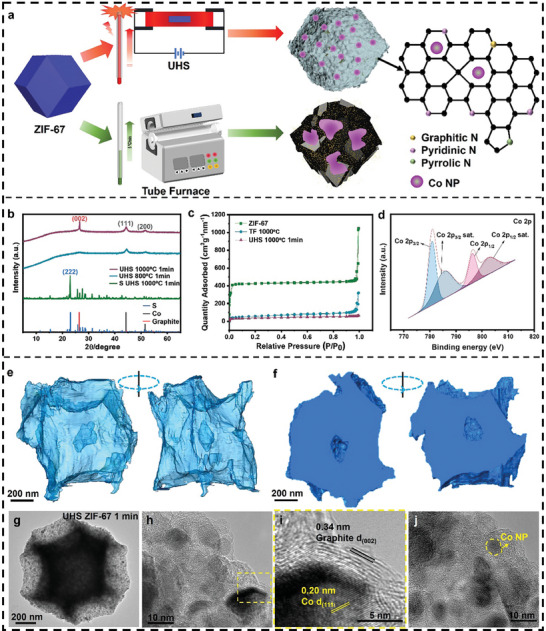
Adjusting Co nanoparticle size and dispersity within carbon nanocage via ultrafast high‐temperature sintering (UHS). a) Schematic diagram of the experimental process for the synthesis of ZIF‐67 and its use of ultra‐high‐speed (UHS) and tube furnace carbonization, as well as the different structures obtained by different sintering methods. b) XRD spectra of UHS sintered ZIF‐67 at different temperatures and times. c) BET test of pristine ZIF‐67 and samples sintered at 1000 °C with tube furnace (TF) and 1000 °C for 1 min with UHS respectively. d) Co XPS spectra of sintered ZIF‐67 at 1000 °C for 1 min with UHS. e) 3D visualization of sintered ZIF‐67 generated from 3D electron tomography. f) Cross section of the sintered ZIF‐67 showing the hollow core within the nanoparticle domain. g–j) The TEM images of ZIF‐67 at different UHS calcination temperatures and times show variations in its morphology.

**Figure** **3**a depicts schematics illustrating the process of preparing ZIF‐67 and fabricating nitrogen‐doped carbon materials through the carbonization of ZIF‐67 using both UHS and traditional furnace methods, highlighting the impact of different sintering techniques on morphology. The crystal structure analysis of ZIF‐67 derivatives, illustrated in Figure 3b, reveals XRD spectra of sintered samples under various rapid heating conditions, all displaying similar diffraction peaks at 2θ = 44° and 51°, corresponding to the (111) and (200) diffraction peaks of Co. The presence of Co NPs was confirmed by XRD after S loading (Figure S12, Supporting Information), which also confirmed the presence of metallic Co nanoparticles. Notably, the sample sintered at UHS 1000 °C for 1 min exhibits a carbon peak at 26.6°, corresponding to the (004) diffraction peak of graphitic carbon, indicating that UHS sintering carbonizes the sample within a mere 1 min. Figure 3c illustrates the surface area characteristics of ZIF‐67 particles and highly nitrogen‐doped carbon nanocages obtained from BET experiments. Analysis of nitrogen adsorption–desorption isotherms reveals a type I isotherm trend for all samples, indicating predominantly microporous structures. Figure S13 (Supporting Information) demonstrates that the sample sintered at UHS 1000 °C for 1 min primarily comprises 1 nm micropores, which are relatively small and effective in immobilizing sulfur and suppressing the shuttle effect of polysulfides during reactions, thereby preventing capacity decay in batteries. X‐ray photoelectron spectroscopy (XPS) characterization was employed to clarify the composition of carbon nanocage. Results show that three types of nitrogen coexist: pyridinic nitrogen, pyrrolic nitrogen, and graphitic nitrogen (Figure S14, Supporting Information). The appearance of pyridinic and pyrrolic nitrogen peaks suggests N–Co bonding and enrichment, indicating the formation of M–N–C coordination (Figures S14 and S15, Supporting Information), serving as active sites for redox reactions. Figure 3d presents the XPS of the Co 2p spectrum of the sample treated at 1000 °C for 1 min by UHS, with characteristic peaks ≈780 and 796 eV attributable to Co nanoparticles. X‐ray photoelectron spectroscopy (XPS) analysis was conducted to determine the elemental composition of samples treated at UHS‐1000 °C for 1 min, UHS‐800 °C for 1 min, and those sintered in a tube furnace. The analysis revealed the nitrogen content and its different types, showing that the sample treated at UHS‐1000 °C for 1 min had the highest pyridinic nitrogen content. This high pyridinic nitrogen concentration potentially provides a large number of active sites. Additionally, from both elemental content and X‐ray diffraction (XRD) data, it is evident that the sample treated at UHS‐1000 °C for 1 min exhibits a higher degree of carbonization and graphitization, with only this sample showing graphite peaks in the XRD pattern. The compositional analysis and XRD characterization indicate that the electrochemical performance of the carbonized ZIF‐67 material depends on factors such as the degree of graphitization, nitrogen doping, and the amount of catalytic Co nanoparticles. The UHS‐1000 °C for 1‐min treatment strikes a balance among these factors, which enhances its electrochemical performance. These structural characteristics are advantageous for their application in lithium–sulfur batteries.

To further characterize the samples after UHS sintering, 3D electron tomography experiments were conducted for structural analysis (Figures S16 and S17, Supporting Information). Typically, TEM images were captured at intervals of 2° by rotating the TEM sample holder to obtain 2D images from multiple angles. These images were aligned and reconstructed into a 3D view using IMOD software,^[^ ^61^, ^62^ ^]^ as depicted in Figure 3e, alongside the cross‐sectional view. The 3D rendering vividly displays the hollow and porous structure within the nanocages (Figure 3f), suggesting that the polyhedral hollow structure remains largely intact following UHS rapid sintering of ZIF‐67. This preservation offers ample space for subsequent sulfur loading (Figures S18 and S19, Movies S7 and S8, Supporting Information).

Likewise, the cross‐sectional view depicted in Figure 3f further underscores the discernible hollow structure, which facilitates sulfur loading. Moreover, HRTEM analysis (Figure 3g–j) reveals the presence of a graphite outer layer with a lattice spacing of 0.34 nm on the sample surface, indicating successful carbonization following short‐term sintering (1 min) (Figure S20, Supporting Information). Additionally, Co nanoparticles with a lattice spacing of 0.20 nm are observed within the carbon layer, suggesting the uniform distribution of numerous nanoscale metallic Co particles, a result that is difficult to achieve using alternative methods.

**Figure 4:**
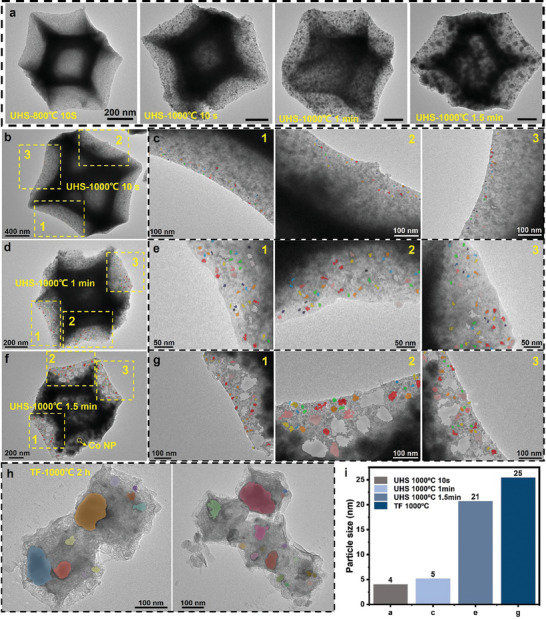
Quantifying Co nanoparticle size and dispersity within carbon nanocage: comparison of different samples generated from UHS, and conventional heating. a) TEM images of sintered ZIF‐67 under different sintering conditions, which shows the Co NP size & dispersity difference. b) TEM image of sintered ZIF‐67 via UHS 1000 °C for 10 s. c) Zoom in TEM images showing the embedded Co NPs within carbon nanocage highlighted in *a*. d) TEM image of sintered ZIF‐67 via UHS 1000 °C for 1 min. e) Zoom in TEM images showing the embedded Co NPs within carbon nanocage highlighted in *c*. f) TEM image of sintered ZIF‐67 via UHS 1000 °C for 1.5 min. g) Zoom in TEM images showing the embedded Co NPs within carbon nanocage which are highlighted in *e*. h) TEM image of sintered ZIF‐67 via TF heating at 1000 °C for 2 h, with zoom in TEM image showing the embedded Co NPs within carbon nanocage which are highlighted in *g*. i) Histogram showing Co NPs average size to compare different sintering methods.

To control the dispersity and size of Co NPs within carbon nanocage, the UHS control experiments were applied. The samples sintered at UHS 1000 °C for different lengths of time were characterized to identify Co NPs size and dispersion (**Figure** **4**a; Figure S21, Supporting Information). To quantify the size of Co NPs size, these TEM images were analyzed via dragonfly tracking with colors showing the Co NPs. Figure 4a–f indicates that, as UHS sintering time prolonged, the nanoparticle size and dispersion changed. Typically, at UHS 1000 °C for 10 s of sintering (Figures S22 and S23, Supporting Information), the Co NPs are ultrasmall which could only be observed in the edge of the nanocage as shown in Figure 4b,c; at UHS 1000 °C for 1 min sintering (Figures S20,S25, and S26, Supporting Information), the Co NPs are ultrasmall with evenly dispersed as shown in Figure 4d,e; at UHS 1000 °C for 1.5 min sintering (Figure S27, Supporting Information), the Co NPs are large as shown in Figure 4f and g. Energy‐dispersive X‐ray spectroscopy (EDX) images of samples treated at UHS‐800 °C for 1 min and UHS‐1000 °C for 1 min reveal that the nitrogen content in the UHS‐1000 °C sample is significantly higher than that in the UHS‐800 °C sample. This observation further confirms the X‐ray photoelectron spectroscopy results. Compared to other sintering conditions, the UHS‐1000 °C for 1‐min treatment achieves an optimal balance among graphitization, nitrogen doping, and the content of catalytic Co nanoparticles. This balance is beneficial for enhancing the electrochemical performance of the material. As a comparison, samples carbonized using traditional tube furnace sintering exhibit significant differences (Figure 4h): the nanocage is structurally damaged and collapsed with huge Co NPs embedded. The Co NP average size was generated from a survey of hundreds of nanoparticles via deep learning analysis using Dragonfly software. As shown in Figure 4i, the ultrasmall Co NPs (4–5 nm) were collected with short UHS sintering time (Figures S28 and S29, Supporting Information), while large Co NPs formed in longer UHS sintering time or traditional heating. Besides the Co NPs size difference, it is clear that its dispersion changed from uniform disperse to uncontrollable bad disperse while sintering time prolonged to hours. It is characterized by revealing a loose and disordered structure with damage to the cage‐like structure (Figures S30–S32, Supporting Information). In conclusion, from a microscopic morphology perspective, ZIF‐67 samples sintered via UHS heating for 1 min at 1000 °C maintain their nanocage, with ultrasmall Co particle size and uniform distribution embedded, and high‐level N‐doping carbon matrix, which provides a novel strategy to create dual doped hard carbon with a simple procedure, shorter synthesis time, higher efficient, and lower energy consumption compared previous reports (Table S1, Supporting Information).

### 2.3 Li–S Batteries Performance via Application of Nanocage as S Carrier

**Figure 5:**
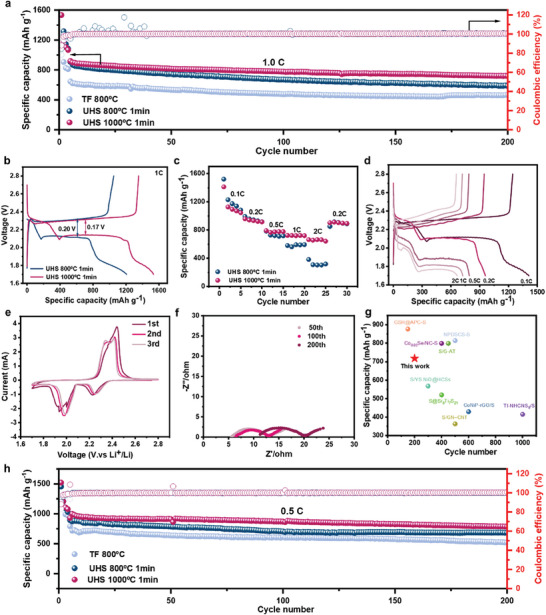
Electrochemical characterization of different sintered ZIF‐67‐based cathodes in Li–S coin cells. a) The cyclic performance of batteries at 1C rate for samples sintered ZIF‐67 via UHS 1000 °C for 1 min, UHS 800 °C for 1 min, and TF 800 °C for 2 h respectively. b) The first cycle discharge/charge curves of batteries at 1C rate for samples sintered ZIF‐67 via UHS 1000 °C for 1 min and UHS 800 °C for 1 min respectively. c) The rate performance of batteries from 0.1C to 2C. d) The discharge/charge voltage curves of the UHS 1000 °C 1 min battery at different rates (0.1C, 0.2C, 0.5C, 1C, and 2C). e) The CV curve of batteries in the first three cycles. f) The AC impedance diagrams of batteries cycled for 50, 100, and 200 cycles. g) Comparison of hard carbon capacity of Li–S batteries with the typical anodes reported previously. h) The cyclic performance of batteries at 0.5C rate for samples sintered ZIF‐67 via UHS 1000 °C for 1 min, UHS 800 °C for 1 min and TF 800 °C for 2 h respectively.

To assess the electrochemical performance of materials obtained via various sintering methods, we constructed pouch‐type Li–S batteries using the obtained carbon materials and conducted performance tests after loading sulfur in the carbon matrix with varying mass quantities (Figure S33, Supporting Information). **Figure** **5**a depicts the charge–discharge cyclic curves of UHS‐sintered ZIF‐67 as sulfur cathode‐limiting materials at a rate of 1C (1 C = 1675 mAh g^−1^) between 1.7 and 2.8 V with activated for the first two cycles at 0.1C followed by the third cycle at 0.2C. After 100 cycles, the discharge‐specific capacity of UHS 1000 °C 1 min was maintained at 777 mAh g^−1^. After 200 cycles, it remained at 718 mAh g^−1^. It is noticeable that even at high rates, the capacity and cycling stability of the battery is well‐preserved, attributed to the confinement effect of the cage‐like porous structure on N1s polysulfides. In Figure 5b, the polarization potentials of UHS 1000 °C 1 min and UHS 800 °C 1 min are 0.17 and 0.20 V, respectively. Lower polarization reflects improved oxidation and reduction kinetics, as well as good ionic conductivity, thereby reducing the decay of the reversible capacity of the battery. Smaller polarization voltage is advantageous for various electrochemical performances. As depicted in Figure 5c, UHS 1000 °C 1 min exhibits commendable rate performance. With the rate increasing from 0.1C to 0.2C, 0.5C, 1, 2C, and 0.2C, the corresponding average discharge specific capacities decrease from 1149, 934, 777, 721, 657 to 902 mAh g^−1^. This finding indicates that N‐doped porous materials derived from ZIF‐67 UHS hold significant potential as high‐rate cathode materials for lithium–sulfur batteries. Figure 5d presents the corresponding discharge/charge voltage curves of the UHS 1000 °C 1 min battery at different rates (0.1C, 0.2C, 0.5C, 1C, and 2C) within the potential range of 1.7–2.8 V. At low rates, the discharge voltage curve exhibits two plateaus characteristic of a typical S cathode (2.3 and 2.1 V), while the charge voltage curve exhibits two plateaus at ≈2.3 and 2.45 V, respectively.

It is noteworthy that at rates exceeding 0.5C, both the discharge plateau and discharge capacity decrease due to high Ohmic and kinetic overpotentials at high rates. However, distinct plateaus are still observed, indicating that UHS 1000 °C 1 min demonstrates high sulfur utilization and excellent rate performance.

The reduction and oxidation peaks on the CV curve (Figure 5e) mirror the reduction and oxidation mechanisms of sulfur during the discharge/charge process. The first peak at 2.25 V corresponds to the reduction of S_8_ to soluble higher‐order Li_2_S_n_ (4≤n≤8), while the second peak at 2.00 V corresponds to the further reaction of higher‐order Li_2_S_n_ to Li_2_S_2_ and eventually to Li_2_S. The cathode oxidation process comprises two stages: the initial oxidation peak corresponds to the formation of Li_2_S_n_, which further oxidizes to S_8_ at 2.42 V; noteworthy is the more pronounced second oxidation peak near 2.42 V, indicating the more complete oxidation of higher‐order Li_2_S_n_ to S_8_ on the composite surface of ZIF‐67 sintered by joule heating. This phenomenon is due to the effective adsorption of Li_2_S_n_ on the material surface and the efficient conversion of Li_2_S_n_ to S_8_ by Co–N bimetallic catalysts. Figure 5h illustrates the cyclic curve of UHS ZIF‐67 as a cathode material at a rate of 0.5C between 1.7 and 2.8 V, demonstrating that after 200 cycles, the discharge‐specific capacity of UHS 1000 °C 1 min was still maintained at 785 mAh g^−1^. The fifth cycle exhibits a capacity of 931 mAh g^−1^, the 100th cycle records a capacity of 870 mAh g^−1^, and the 200th cycle maintains a capacity of 785 mAh g^−1^. The capacity retention rates from the fifth to the hundredth cycle are 93.4%, and from the fifth to the two hundredth cycle are 84.3% (Figure S34, Supporting Information). Figure 5f presents impedance plots depicting the impedance at the 50th, 100th, and 200th cycles of battery operation. With increasing cycle numbers, the impedance also rises, accompanied by a certain degree of capacity decay. However, the capacity decay is relatively minimal, indicating good interface stability of the electrode material. The post‐cycling interface analysis of the battery (Figure S35, Supporting Information) supports the same conclusion. The interface thickness of the battery exhibits no significant change before and after cycling, affirming that UHS ZIF‐67, as a cathode material for Li–S batteries, offers protection for long‐term battery cycling through the confinement effect of its nanocages. The outstanding performance observed exceeds that of both the control sample hard carbon and the latest cathode materials for Li–S batteries, as depicted in Figure 5g ^[^ ^63^, ^64^, ^65^, ^66^, ^67^, ^68^, ^69^, ^70^, ^71^ ^]^ and detailed in Table S2 (Supporting Information) of the Supporting Information. This superiority can be ascribed to the combination of ultrasmall cobalt nanoparticles and nitrogen doping in hard carbon, which optimizes the electrolyte/electrode interface, promoting efficient charge transfer and reducing the polarization effects commonly encountered in Li–S batteries.

Performance tests of the batteries indicate that different sintering methods significantly impact the performance of the electrode materials. The sample sintered at UHS‐1000 °C for 1 min achieves an optimal balance among graphitization, nitrogen doping, and Co nanoparticle content. This balance enables the material to exhibit superior performance under high currents, broad temperature ranges, and long cycling conditions compared to traditional tube furnace‐sintered samples (Figure 5h). This highlights the importance of establishing a balanced internal structure in functional materials where multiple factors are controlled.

### 2.4 Mechanism of Enhanced Li–S Batteries Performance

**Figure 6:**
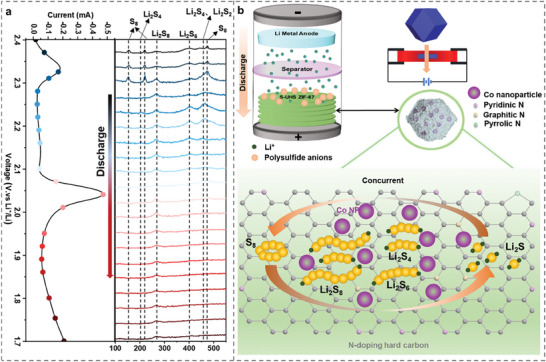
Mechanistic comparison of Li–S redox reactions at a Sulphur (S) cathode with and without Co NP catalysis. (a) The in situ Raman spectra of Li–S battery. (b) The schematics illustrate the proposed mechanism by which UHS sintered ZIF‐67 enhances the electrochemical performance of lithium–sulfur batteries due to ultrasmall Co NP catalysis.

Drawing from material characterizations and battery performance tests, the proposed mechanism elucidating the enhanced performance of UHS‐sintered ZIF‐67 in Li‐S batteries can be delineated as follows (**Figure** **6**, Note S4, Supporting Information), which is a concurrent process: i) Confinement Effect: The cage‐like porous structure of the carbonized ZIF‐67, derived from UHS, offers efficient confinement for sulfur and polysulfide species during the charge–discharge process. This confinement effectively curtails the dissolution and shuttle effect of polysulfides (Figure S36, Supporting Information),^[^ ^72^, ^73^ ^]^ enhancing the stability and cycling performance of the Li–S battery.^[^ ^74^ ^]^ ii) Augmented Catalytic Activity: Within the carbonized ZIF‐67 material, uniformly dispersed ultrasmall Co nanoparticles (diameter size < 8 nm) are present. These Co nanoparticles serve as effective catalysts, facilitating the conversion of polysulfides^[^ ^75^, ^76^ ^]^ in concurrent rather than stepwise (Figure 6a,b). Typically, analysis of the in situ Raman spectra reveals that peaks at 152, 218, and 474 cm⁻¹ are attributable to S₈, while the peak at 265 cm⁻¹ corresponds to Li₂S₈. As the reaction progresses, a peak ≈400 cm⁻¹ becomes increasingly prominent, indicating the formation of Li₂S₆. During the voltage reduction process, the appearance of peaks at 455 and 203 cm⁻¹ is due to the conversion of Li₂S₆ to Li₂S₄ and Li₂S₂. When the voltage is reduced to 1.7 V, almost all peaks corresponding to lithium polysulfides disappear, indicating that They promote the transformation of higher‐order polysulfides into lower‐order counterparts during discharge and impede the reverse reaction during charge, thereby amplifying the battery's efficiency and longevity.^[^ ^75^, ^77^ ^]^ The boosted catalytic activity plays a pivotal role in elevating the electrochemical performance of the Li–S battery. iii) Ensuring Electrochemical Interface Stability: The nitrogen‐doped carbon matrix originating from UHS ZIF‐67 furnishes active sites essential for the adsorption and stabilization of polysulfide intermediates.^[^ ^78^, ^79^ ^]^ Analysis of the nitrogen content in the XPS spectra (Figure S15, Supporting Information) and EDX spectra (Figure S20, Supporting Information) shows that the sample sintered at UHS‐1000 °C for 1 min has higher nitrogen doping and pyridinic nitrogen content compared to samples obtained under other experimental conditions. This stabilization inhibits their diffusion and deposition onto the lithium anode, thereby reducing the formation of lithium dendrites and enhancing the long‐term stability of the Li–S battery. iv) Enhanced Electron and Ion Transport: The porous configuration of the carbonized ZIF‐67 material facilitates electron and lithium‐ion movement within the electrode throughout charge–discharge cycles.^[^ ^80^, ^81^, ^82^ ^]^ This enhanced transport kinetics minimize polarization effects, thereby boosting the overall efficiency of the battery.

## 3 Conclusion

In summary, this study presents a thorough exploration using in situ liquid‐phase TEM to unravel ZIF‐67 growth dynamics. First, the in situ liquid‐phase TEM observations offer valuable insights into the growth mechanism of ZIF‐67 nanoparticles, unveiling a dynamic process involving both classical crystallization and nonclassical crystallization mechanisms,^[^ ^14^, ^50^, ^83^ ^]^ including liquid–liquid phase separation, nanoparticle attachment, and self‐assembly. This comprehension of ZIF‐67 growth kinetics is pivotal for tailoring the material's properties to suit specific application requirements. Second, the UHS process enables the rapid synthesis of ZIF‐67 derivatives with unique structural features, including a porous carbon matrix with embedded ultrasmall Co nanoparticles. These features, alongside nitrogen doping, enhance electrochemical performance by facilitating sulfur confinement in Li–S batteries. Overall, the synergistic integration of in situ liquid‐phase TEM studies and the application of UHS ZIF‐67 in Li–S batteries offers a comprehensive approach to comprehending the growth dynamics of MOF‐derived materials and harnessing their distinct properties for advanced energy storage.^[^ ^84^, ^85^ ^]^

## Conflict of Interest

The authors declare no conflict of interest.

## Author Contributions

X.H.S. raised the idea, conducted the experiments, and led the project. R.H. and X.H.S. performed the in situ TEM experiments, MOF synthesis, and UHS sinter. X.Y.Z. and R.H. did the in situ TEM movies analysis. S.K., D.J., and J.K. did 3D reconstruction and segmentation. X. H. Song and E. H. Ang performed the related analysis of 3D structures. R.H., Q.H., and X.F. did the Li–S battery test. X.H.S., X.S., and H.F.X. made discussions, and funding acquisitions, led supervision, provided helpful advice, and revised the manuscript. All authors have approved the final version of the manuscript.

## Supporting information



Supporting Information

Supplementary Movie 1

Supplementary Movie 2

Supplementary Movie 3

Supplementary Movie 4

Supplementary Movie 5

Supplementary Movie 6

Supplementary Movie 7

Supplementary Movie 8

## Data Availability

The data that support the findings of this study are available in the supplementary material of this article.

## References

[1] J. Lee , O. K. Farha , J. Roberts , K. A. Scheidt , S. T. Nguyen , J. T. Hupp , Chem. Soc. Rev. 2009, 38, 1450. 19384447 10.1039/b807080f

[2] H. Furukawa , K. E. Cordova , M. O'Keeffe , O. M. Yaghi , Science 2013, 341, 1230444. 23990564 10.1126/science.1230444

[3] H. Zhong , M. Wang , G. Chen , R. Dong , X. Feng , ACS Nano 2022, 16, 1759. 35049290 10.1021/acsnano.1c10544

[4] L. Ma , H. Chen , J. Wu , Y. Lv , X. Chen , X. Li , Q. J. Li , J. Di , Y. Chen , Adv. Energy Mater. 2022, 12, 2103152.

[5] B. Sun , Z. Sun , Y. Yang , X. L. Huang , S. C. Jun , C. Zhao , J. Xue , S. Liu , H. K. Liu , S. X. Dou , ACS Nano 2023, 18, 28. 38117556 10.1021/acsnano.3c08240

[6] K. Jayaramulu , S. Mukherjee , D. M. Morales , D. P. Dubal , A. K. Nanjundan , A. Schneemann , J. Masa , S. Kment , W. Schuhmann , M. Otyepka , Chem. Rev. 2022, 122, 17241. 36318747 10.1021/acs.chemrev.2c00270 PMC9801388

[7] Y. Bai , C. Liu , Y. Shan , T. Chen , Y. Zhao , C. Yu , H. Pang , Adv. Energy Mater. 2022, 12, 2100346.

[8] F. Qi , Z. Sun , X. Fan , Z. Wang , Y. Shi , G. Hu , F. Li , Adv. Energy Mater. 2021, 11, 2100387.

[9] C. Zhou , Z. Li , X. Xu , L. Mai , Natl. Sci. Rev. 2021, 8, nwab055. 34987837 10.1093/nsr/nwab055 PMC8692935

[10] X. Chen , M.‐x. Li , J.‐l. Yan , L.‐l. Zhang , New Carbon Mater. 2024, 39, 78.

[11] B. P. Carpenter , A. R. Talosig , B. Rose , G. Di Palma , J. P. Patterson , Chem. Soc. Rev. 2023, 52, 6918. 37796101 10.1039/d3cs00312d

[12] R. Dhaoui , S. L. Cazarez , L. Xing , E. Baghdadi , J. T. Mulvey , N. S. Idris , P. J. Hurst , M. P. Vena , G. D. Palma , J. P. Patterson , Adv. Funct. Mater. 2024, 34, 2312972.

[13] J. Xing , L. Schweighauser , S. Okada , K. Harano , E. Nakamura , Nat. Commun. 2019, 10, 3608. 31444338 10.1038/s41467-019-11564-4 PMC6707309

[14] X. Liu , S. W. Chee , S. Raj , M. Sawczyk , P. Král , U. Mirsaidov , Proc. Natl. Acad. Sci. USA 2021, 118, e2008880118. 33649204 10.1073/pnas.2008880118 PMC7958460

[15] J. P. Patterson , P. Abellan , M. S. Denny Jr , C. Park , N. D. Browning , S. M. Cohen , J. E. Evans , N. C. Gianneschi , J. Am. Chem. Soc. 2015, 137, 7322. 26053504 10.1021/jacs.5b00817

[16] J. Korpanty , N. C. Gianneschi , Acc. Chem. Res. 2023, 56, 2298. 37580021 10.1021/acs.accounts.3c00211

[17] L. Kollias , D. C. Cantu , M. A. Tubbs , R. Rousseau , V. A. Glezakou , M. Salvalaglio , J. Am. Chem. Soc. 2019, 141, 6073. 30887804 10.1021/jacs.9b01829

[18] I. E. Khalil , J. Fonseca , M. R. Reithofer , T. Eder , J. M. Chin , Coord. Chem. Rev. 2023, 481, 215043.

[19] L. Kollias , R. Rousseau , V. A. Glezakou , M. Salvalaglio , J. Am. Chem. Soc. 2022, 144, 11099. 35709413 10.1021/jacs.1c13508

[20] A. V. Dighe , L. Huelsenbeck , R. R. Bhawnani , P. Verma , K. H. Stone , M. R. Singh , G. Giri , JACS Au 2022, 2, 453. 35252994 10.1021/jacsau.1c00494 PMC8889615

[21] D. J. Lewis , L. Z. Zornberg , D. J. Carter , R. J. Macfarlane , Nat. Mater. 2020, 19, 719. 32203459 10.1038/s41563-020-0643-6

[22] S. Yuan , L. Feng , K. Wang , J. Pang , M. Bosch , C. Lollar , Y. Sun , J. Qin , X. Yang , P. Zhang , Adv. Mater. 2018, 30, 1870277. 10.1002/adma.201704303 29430732

[23] J. J. De Yoreo , P. U. Gilbert , N. A. Sommerdijk , R. L. Penn , S. Whitelam , D. Joester , H. Zhang , J. D. Rimer , A. Navrotsky , J. F. Banfield , Science 2015, 349, aaa6760. 26228157 10.1126/science.aaa6760

[24] D. Kashchiev , J. Chem. Phys. 2003, 118, 1837.

[25] X. G. Wang , Q. Cheng , Y. Yu , X. Z. Zhang , Angew. Chem., Int. Ed. 2018, 57, 7836. 10.1002/anie.201803766 29700914

[26] F. Millange , M. I. Medina , N. Guillou , G. Férey , K. M. Golden , R. I. Walton , Angew. Chem. 2010, 122, 775. 10.1002/anie.200905627 20017176

[27] A. F. Ogata , A. M. Rakowski , B. P. Carpenter , D. A. Fishman , J. G. Merham , P. J. Hurst , J. P. Patterson , J. Am. Chem. Soc. 2020, 142, 1433. 31913610 10.1021/jacs.9b11371

[28] S. L. Anderson , A. Gładysiak , P. G. Boyd , C. P. Ireland , P. Miéville , D. Tiana , B. Vlaisavljevich , P. Schouwink , W. Van Beek , K. J. Gagnon , CrystEngComm 2017, 19, 3407.

[29] L. Kuhrts , S. Prévost , D. M. Chevrier , P. Pekker , O. Spaeker , M. Egglseder , J. Baumgartner , M. Pósfai , D. Faivre , J. Am. Chem. Soc. 2021, 143, 10963. 34264055 10.1021/jacs.1c02687 PMC8323100

[30] M. Filez , C. Caratelli , M. Rivera‐Torrente , F. Muniz‐Miranda , M. Hoek , M. Altelaar , A. J. Heck , V. Van Speybroeck , B. M. Weckhuysen , Cell Rep. Phys. Sci. 2021, 2, 100680.

[31] D. Salionov , O. O. Semivrazhskaya , N. P. Casati , M. Ranocchiari , S. Bjelić , R. Verel , J. A. van Bokhoven , V. L. Sushkevich , Nat. Commun. 2022, 13, 3762. 35768412 10.1038/s41467-022-31294-4 PMC9243051

[32] F. H. Larsen , J. Skibsted , H. J. Jakobsen , N. C. Nielsen , J. Am. Chem. Soc. 2000, 122, 7080.

[33] R. S. Madsen , A. Qiao , J. Sen , I. Hung , K. Chen , Z. Gan , S. Sen , Y. Yue , Science 2020, 367, 1473. 32217725 10.1126/science.aaz0251 PMC7325427

[34] N. Yuan , T. L. Church , E. G. Brandt , N. Hedin , X. Zou , D. Bernin , Sci. Rep. 2018, 8, 17530. 30510207 10.1038/s41598-018-35842-1 PMC6277383

[35] W. Liang , H. Xu , F. Carraro , N. K. Maddigan , Q. Li , S. G. Bell , D. M. Huang , A. Tarzia , M. B. Solomon , H. Amenitsch , J. Am. Chem. Soc. 2019, 141, 2348. 30636404 10.1021/jacs.8b10302

[36] K. I. Hadjiivanov , D. A. Panayotov , M. Y. Mihaylov , E. Z. Ivanova , K. K. Chakarova , S. M. Andonova , N. L. Drenchev , Chem. Rev. 2020, 121, 1286. 33315388 10.1021/acs.chemrev.0c00487

[37] C. L. Jones , C. E. Hughes , H. H.‐M. Yeung , A. Paul , K. D. Harris , T. L. Easun , Chem. Sci. 2021, 12, 1486. 10.1039/d0sc04892e PMC8179150 34163912

[38] J. Cravillon , C. A. Schröder , R. Nayuk , J. Gummel , K. Huber , M. Wiebcke , Angew. Chem. Int. Ed. Engl. 2011, 50, 8067. 21748830 10.1002/anie.201102071

[39] S. Saha , M. Wiebcke , K. Huber , Cryst. Growth Des. 2018, 18, 4653.

[40] S. Y. Chen , W. S. Lo , Y. D. Huang , X. Si , F. S. Liao , S. W. Lin , B. P. Williams , T. Q. Sun , H. W. Lin , Y. An , Nano Lett. 2020, 20, 6630. 32786948 10.1021/acs.nanolett.0c02265

[41] Y. Qian , F. Zhang , S. Zhao , C. Bian , H. Mao , D. J. Kang , H. Pang , Nano Energy 2023, 111, 108415.

[42] S. Guo , M. Gao , W. Zhang , F. Liu , X. Guo , K. Zhou , Adv. Mater. 2023, 35, 2303065. 10.1002/adma.202303065 37319033

[43] Y. J. Tang , H. Zheng , Y. Wang , W. Zhang , K. Zhou , Adv. Funct. Mater. 2021, 31, 2102648. 10.1002/adfm.202007017 PMC9273013 35822179

[44] B. Y. Xia , Y. Yan , N. Li , H. B. Wu , X. W. D. Lou , X. Wang , Nat. Energy 2016, 1, 15006.

[45] Y. Chen , T. Wang , H. Tian , D. Su , Q. Zhang , G. Wang , Adv. Mater. 2021, 33, 2003666. 10.1002/adma.202003666 34096100

[46] Q. Pang , X. Liang , C. Y. Kwok , L. F. Nazar , Nat. Energy 2016, 1, 16132.

[47] L. Yu , H. Hu , H. B. Wu , X. W. Lou , Adv. Mater. 2017, 29, 1604563. 10.1002/adma.201604563 28092123

[48] Z. Li , L. Sun , K. Wang , Y. Zhang , Mater. Today Sustain. 2023, 22, 100392.

[49] Y. Yang , B. Sun , Z. Sun , J. Xue , J. He , Z. Wang , K. Sun , Z. Sun , H. K. Liu , S. X. Dou , Coord. Chem. Rev. 2024, 510, 215836.

[50] M. J. Van Vleet , T. Weng , X. Li , J. Schmidt , Chem. Rev. 2018, 118, 3681. 29514005 10.1021/acs.chemrev.7b00582

[51] O. Shekhah , H. Wang , D. Zacher , R. A. Fischer , C. Wöll , Angew. Chem., Int. Ed. 2009, 48, 5038. 10.1002/anie.200900378 19492375

[52] J. Li , F. L. Deepak , Chem. Rev. 2022, 122, 16911. 36347015 10.1021/acs.chemrev.1c01067

[53] S. Karthika , T. Radhakrishnan , P. Kalaichelvi , Cryst. Growth Des. 2016, 16, 6663.

[54] S. L. Hanna , M. Barsoum , T. T. Debela , C. D. Malliakas , M. A. Gaidimas , J. G. Knapp , K. O. Kirlikovali , C. H. Hendon , V. P. Dravid , O. K. Farha , ACS Mater. Lett. 2023, 5, 2518.

[55] Y. Sun , X. Zhang , R. Huang , D. Yang , J. Kim , J. Chen , E. H. Ang , M. Li , L. Li , X. Song , Nanoscale 2024, 16, 2945. 38236129 10.1039/d3nr04480g

[56] C. Liu , L. Yao , Q. Chen , Microsc. Microanal. 2022, 28, 94.

[57] L. Yao , Q. Chen , Intelligent Nanotechnology, Elsevier, Amsterdam 2023, 246, p. 279.

[58] Y. Li , K. Wang , W. Zhou , Y. Li , R. Vila , W. Huang , H. Wang , G. Chen , G. H. Wu , Y. Tsao , Matter 2019, 1, 428. 34104881 10.1016/j.matt.2019.06.001 PMC8184120

[59] L. Liu , D. Zhang , Y. Zhu , Y. Han , Commun. Chem. 2020, 3, 99. 36703329 10.1038/s42004-020-00361-6 PMC9814830

[60] J. Zhang , N. Cheng , B. Ge , Adv. Phys.: X 2022, 7, 2046157.

[61] X. Song , J. W. Smith , J. Kim , N. J. Zaluzec , W. Chen , H. An , J. M. Dennison , D. G. Cahill , M. A. Kulzick , Q. Chen , ACS Appl. Mater. Interfaces 2019, 11, 8517. 30676014 10.1021/acsami.8b20826

[62] J. R. Kremer , D. N. Mastronarde , J. R. McIntosh , J. Struct. Biol. 1996, 116, 71. 8742726 10.1006/jsbi.1996.0013

[63] J. Liu , M. Xue , Y. Zhou , S. Liu , T. Yan , Chem. Eng. J. 2023, 459, 141556.

[64] H. J. Peng , J. Q. Huang , M. Q. Zhao , Q. Zhang , X. B. Cheng , X. Y. Liu , W. Z. Qian , F. Wei , Adv. Funct. Mater. 2014, 24, 2772.

[65] Z. Yu , M. Liu , D. Guo , J. Wang , X. Chen , J. Li , H. Jin , Z. Yang , X. a. Chen , S. Wang , Angew. Chem. 2020, 132, 6468. 10.1002/anie.201914972 31971656

[66] J. Wang , H. Yang , Z. Chen , L. Zhang , J. Liu , P. Liang , H. Yang , X. Shen , Z. X. Shen , Adv. Sci. 2018, 5, 1800621. 10.1002/advs.201800621 PMC6247042 30479918

[67] Y. Xie , J. Cao , X. Wang , W. Li , L. Deng , S. Ma , H. Zhang , C. Guan , W. Huang , Nano Lett. 2021, 21, 8579. 34652920 10.1021/acs.nanolett.1c02037

[68] T. Xiao , F. Yi , M. Yang , W. Liu , M. Li , M. Ren , X. Zhang , Z. Zhou , J. Mater. Chem. A 2021, 9, 16692.

[69] Y. Wu , D. Li , J. Pan , Y. Sun , W. Huang , M. Wu , B. Zhang , F. Pan , K. Shi , Q. Liu , J. Mater. Chem. A 2022, 10, 16309.

[70] D. Yang , Y. Han , M. Li , C. Li , W. Bi , Q. Gong , J. Zhang , J. Zhang , Y. Zhou , H. Gao , Adv. Funct. Mater. 2024, 2401577, 10.1002/adfm.202401577

[71] Z. Zhang , L. L. Kong , S. Liu , G. R. Li , X. P. Gao , Adv. Energy Mater. 2017, 7, 1602543.

[72] Y. Huang , L. Lin , C. Zhang , L. Liu , Y. Li , Z. Qiao , J. Lin , Q. Wei , L. Wang , Q. Xie , Adv. Sci. 2022, 9, 2106004. 10.1002/advs.202106004 PMC9036004 35233996

[73] W. Ren , W. Ma , S. Zhang , B. Tang , Energy Storage Mater. 2019, 23, 707.

[74] Q. He , W. Chen , B. Fan , Q. Wei , Y. Zou , Chem. Eng. J. 2024, 496, 153813.

[75] W. Xu , S. Lang , K. Wang , R. Zeng , H. Li , X. Feng , M. R. Krumov , S. M. Bak , C. J. Pollock , J. Yeo , Sci. Adv. 2023, 9, eadi5108. 37585528 10.1126/sciadv.adi5108 PMC10431713

[76] S. Lang , S. H. Yu , X. Feng , M. R. Krumov , H. D. Abruña , Nat. Commun. 2022, 13, 4811. 35973986 10.1038/s41467-022-32139-w PMC9381601

[77] X. Zhou , R. Meng , N. Zhong , S. Yin , G. Ma , X. Liang , Small Methods 2021, 5, 2100571. 10.1002/smtd.202100571 34927940

[78] L. C. Yin , J. Liang , G. M. Zhou , F. Li , R. Saito , H. M. Cheng , Nano Energy 2016, 25, 203.

[79] Q. Chen , S. Chen , J. Ma , S. Ding , J. Zhang , Adv. Funct. Mater. 2024, 34, 2308272.

[80] L. Du , B. Zhang , W. Deng , Y. Cheng , L. Xu , L. Mai , Adv. Energy Mater. 2022, 12, 2200501.

[81] H. Xu , A. Manthiram , Nano Energy 2017, 33, 124.

[82] L. Jin , J. Chen , Z. Fu , X. Qian , J. Cheng , Q. Hao , K. Zhang , Sustain. Mater. Technol. 2023, 35, e00571.

[83] J. P. Patterson , P. Abellan , M. S. Denny Jr , C. Park , N. D. Browning , S. M. Cohen , J. E. Evans , N. C. Gianneschi , J. Am. Chem. Soc. 2015, 137, 7322. 26053504 10.1021/jacs.5b00817

[84] C. Zhao , F. Huo , Y. Yang , J. Ruan , F. Chai , H. Xu , Y. Liu , L. Zhang , A. Cabot , Z. Sun , Adv. Funct. Mater. 2024, 34, 2402175.

[85] Y. Jiang , M. Du , P. Geng , B. Sun , R. Zhu , H. Pang , J. Colloid Interface Sci. 2024, 664, 617. 38490037 10.1016/j.jcis.2024.03.015

