# Supplementary material for: Unveiling the Dynamic Pathways of Metal–Organic Framework Crystallization and Nanoparticle Incorporation for Li–S Batteries
Source: Adv Sci (Weinh). 2024 Sep 24;11(43):2407984. doi: 10.1002/advs.202407984 (PMC11578323; doi:10.1002/advs.202407984)
Supplement: Supplementary file 1 — Supporting Information [file ADVS-11-2407984-s002.docx]

Supplementary Materials for

**Unveiling the Dynamic Pathways of Metal-Organic Framework Crystallization and Nanoparticle Incorporation for Li-S Batteries**

Xiaohui Song^1,7,*^, Rui Huang^1^, Xingyu Zhang^2.*^, Qiang Chang^1^, Semi Kim^3,4^, Daeun Jeong^3,4^, Qian Hou^1^, Juyeong Kim^3,4^, Edison Huixiang Ang^5^, Xiaowei Su^6^, Xuyong Feng^1^, Hongfa Xiang ^1,7,*^

*^1^School of Materials Science and Engineering, Hefei University of Technology, Anhui Province, 230009, P.R. China*

*^2^School of Mathematics, Statistics and Mechanics, Beijing University of Technology, Beijing 100124, P.R. China*

*^3^Department of Chemistry and Research Institute of Natural Sciences, Gyeongsang National University, Jinju 52828, South Korea*

*^4^Research Institute of Advanced Chemistry, Gyeongsang National University, Jinju 52828, South Korea*

*^5^Natural Sciences and Science Education, National Institute of Education, Nanyang Technological University, Singapore 637616, Singapore*

*^6^Anhui Honghai New Materials Co., Ltd, Anqing City, Anhui Province, 246100, P.R. China*

*^7^Engineering Research Center of High Performance Copper Alloy Materials and Processing, Ministry of Education, Hefei University of Technology, Hefei 230009, China*

^*^Corresponding authors’ address: [xiaohuisong@hfut.edu.cn](mailto:xiaohuisong@hfut.edu.cn); xingyu0711@bjut.edu.cn; [hfxiang@hfut.edu.cn](mailto:hfxiang@hfut.edu.cn);

**This file includes:**

Chemicals

Experimental and methods

Supplementary Notes 1-4

Supplementary Fig. S1-36

Supplementary Table S1-3

Legends for Movies S1-8

References

**Experimental and Characterization**

Chemicals. Sulfuric acid (H_2_SO_4_, Analytical Pure (AR)); sublimed sulfur (Analytical Pure (AR)); hydrochloric acid (HCl, Analytical Pure (AR)); cobalt acetate tetrahydrate ((CH_3_COO)_2_Co·_4_H_2_O, Analytical Pure (AR)); ethanol (EtOH, Analytical Pure (AR)) all from China National Pharmaceutical Group Corporation; 2-methylimidazole (C_4_H_6_N_2_, Alfa Aesar, 98%); Ketjenblack (Kelude, Analytical Pure (AR)); 1-methyl-2-pyrrolidinone (C_5_H_9_NO, Aladdin, Analytical Pure (AR)); Polyvinylidene Fluoride (-(C_2_H_2_F_2_)n-, Kelude, Analytical Pure (AR)). All reagents and chemicals were used without further purification in this work.

ZIF-67 preparation. 0.6 g of cobalt acetate tetrahydrate was dissolved in 5 ml of deionized water and sonicated for 2 minutes until the solid completely dissolved to obtain solution A. Subsequently, 2.24 g of 2-methylimidazole (2-MIM) was dissolved in 5 ml of deionized water and sonicated for 2 minutes until the solid fully dissolved to obtain solution B. Solution A was then poured into solution B, rapidly stirred for 20 seconds at room temperature, and left to stand for 24 hours. Afterward, the supernatant was removed using a pipette, and the solid was washed with ethanol and deionized water by centrifugation (8000 rpm/min - three times). Finally, the solid was dried in a 55 °C oven for 8 hours to obtain a blue powder.

UHS ZIF-67 experiment. After drying, 1.2 g of ZIF-67 was placed into a UHS apparatus under an argon atmosphere. The temperature was rapidly increased to 650 °C, 800 °C, or 1000 °C and maintained for 1 minute, 1.5 minutes, 10 seconds and 20 seconds, respectively, to adjust the sintering conditions. Subsequently, the sample was allowed to cool naturally to obtain nitrogen-doped graphite material.

Graphene liquid cell preparation and in-situ liquid-phase TEM experiments. The graphene liquid cell preparation was conducted following a previous method with some modifications. Two graphene-coated TEM grids were taken and placed graphene side up on a glass slide. Using a small surgical scalpel blade, the edge of one of the graphene-coated TEM grids was cut off, approximately 1/4 of the area of the grid. The in-situ liquid-phase TEM imaging experiments were conducted in a 120 kV TEM-1400 Flash TEM (JEOL Ltd., Tokyo, Japan) equipped with a Gatan camera (Gatan Inc., Pleasanton, CA, USA). The in-situ image series were acquired at a rate of 5 frames per second and an incident electron flux of <2.0 e^–^ Å^–2^ s^–1^.

In-Situ Liquid-Phase TEM Study on ZIF-67 Growth Mechanism. 0.48 g of cobalt(II) acetate tetrahydrate and 1.8 g of 2-methylimidazole were separately dissolved in 5 ml of water, denoted as solutions A and B, respectively. Then, 100 μL of solution A was added to 80 μL of water followed by the addition of 100 μL of solution B to obtain solution C. Subsequently, 0.35 μL of solution C was drop-cast onto the center of a copper grid, covered with another copper grid, and placed onto the TEM sample holder for observation. The D/MAX2500VL/PC X-ray diffractometer produced by Rigaku (Nippon Institute of Science) was used to characterize the crystal structure and phase composition in this experiment. The scanning range was 10-90°, and the scanning speed was 10° min^-1^. HRTEM testing was conducted using a transmission electron microscope (TEM) produced by Hitachi Company in Japan, model JEM-2100F and Talos F200 XG2. The in-situ TEM experiments were conducted on JEM 1400. The SEM model used in this paper is the Gemini model produced by Zeiss. The X-ray photoelectron spectroscopy (XPS) model ESCLAB250Xi produced by Thermo Fisher Technology in the United States was used to analyze the chemical states.

Electrochemical performance evaluation. This experiment utilized the Neware battery testing system to assess the battery's cycling and rate performance, with an operating voltage range of 1.7-2.8 V. Cycling tests involved preconditioning with 2 cycles at 0.1 C (1 C = 1675 mAg^-1^), followed by 2 cycles at 0.2 C. Rate performance tests consisted of continuous cycling at 0.1 C, 0.2 C, 0.5 C, 1 C, 2 C, and 0.2 C currents for 5 cycles each. Electrochemical characterization involved cyclic voltammetry (CV) testing and electrochemical impedance spectroscopy (EIS) testing using a CHI660E electrochemical workstation within a voltage window of 1.7-2.8 V and frequency range of 1000 kHz to 0.1 Hz, respectively. Constant current charge/discharge measurements were conducted between 1.7 V and 2.8 V using a battery testing station (Land T2001A). All electrochemical tests were performed at 25°C. Details regarding Li-S battery assembly can be found in the supplementary information (SI).

Experimental methods

ZIF-67 sintering in tube furnace. In the process of preparing N-doped composite porous carbon materials using ZIF-67 as a precursor through high-temperature sintering, argon atmosphere was chosen for sintering. ZIF-67 weighing 500 mg was sintered at three comparative temperatures of 650 °C, 800 °C, and 1000 °C respectively. The heating rate was uniformly set at 2 °C/min, and the dwell time was uniformly set at 2 hours.

Large Co particle etching. After grinding 160 mg of the carbonized sample (sintered at 1000 °C, 2h), it was added to 10 ml of 3 M H_2_SO_4_ and allowed to etch for 8 hours. Upon completion of the reaction, the sample was centrifuged and washed three times with water to remove residual chemicals and acid.

S loading. The carbon-sulfur mixture with a mass ratio of 2:3 was added to a sealed reaction vessel and heated in a tube furnace at a rate of 5 °C/min until reaching 155 °C. It was maintained at this temperature for 12 hours under an argon atmosphere. After sintering, the furnace was allowed to cool to room temperature, and the sample was removed, ground, and placed in a ceramic boat. The temperature was then raised to 200 °C at a rate of 5 °C/min and held for 2 hours before cooling down to room temperature along with the furnace.

Preparation of cathode electrode. After mixing the sulfur-loaded sample, the conductive agent Ketjenblack (KB), and the binder PVDF in a ratio of 7:2:1, add an appropriate amount of NMP for ball milling. The resulting slurry is uniformly coated onto aluminum foil with a carbon layer. Finally, the coated foil is dried at 55°C to obtain the cathode electrode plate.

The assembly of CR2032 button cell batteries. The assembly process of the half-cell batteries described in this paper was completed in a glovebox with oxygen levels below 0.01 ppm. The assembly sequence is as follows: negative electrode shell, lithium metal foil, 15 µL electrolyte, separator, 15 µL electrolyte, negative electrode foil, spacer, spring, positive electrode shell. The assembled battery was immediately sealed using a sealing machine. Subsequently, the sealed battery was removed from the glovebox and allowed to stand for at least 8 hours at 25°C for subsequent electrochemical testing. The lithium metal foil was purchased from SciLab, and the separator used was a polypropylene separator purchased from Celgard, USA. The electrolyte used was 1.0 M LiTFSI (Bistrifluoromethanesulfonimide lithium salt) in DOL(C₃H₆O₂):DME(C_4_H_10_O_2_)=1:1 VOL% with 2.0 wt% LiNO_3_.

**TEM grids liquid cell sample preparation**

1. Preparation of in-situ liquid-phase TEM solution: 0.48 g of cobalt(II) acetate tetrahydrate and 1.8 g of 1,3-dimethylimidazolium were separately added into 5 ml of water, denoted as solutions A and B, respectively. 100 μL of solution A was added into 80 μL of water followed by the addition of 100 μL of solution B to obtain the in-situ liquid-phase TEM solution.
2. Normally, the sample loading procedure is as follows: Take two pure carbon-coated TEM grids (Buy 200 mesh pure carbon film copper net won by Guangzhou.) and place them graphene side up on a glass slide. Place 0.35 µL droplet of solution to be encapsulated on the non-cut graphene-coated TEM grid center. Use a tweezer to hold the edge of the TEM grid down while placing the droplet so that the capillary forces do not pick up the TEM grid.
3. Quickly and carefully place the pure carbon-coated TEM grid with the cut corner on top of the droplet; the goal is to have the second grid come to rest on top of the first grid with no liquid getting squeezed out.
4. Wait 5 min to let liquid cell pockets form: Some evaporation of the liquid may occur as the pockets are forming, but once a hermetic seal is formed, no additional liquid loss is likely.
5. Place the liquid cell in a traditional TEM single tilt holder, and Load the TEM holder into the TEM column. Then, use the nanoparticles and amorphous carbon in the sample to properly align the TEM beam, and image. Finally, remove the holder from the beam path and calibrate electron beam dose rate.
6. After the assembly, the liquid cell is put into a vacuum tank for sample transfer (also help to check the liquid leaking) and then quickly put into a TEM sample room, and the whole process minimizes the contact time with air.

**Image processing via deep learning (movies analysis)**

TEM (Transmission Electron Microscopy) movie processing analysis using Dragonfly deep learning involves employing advanced artificial intelligence techniques to extract valuable insights and information from dynamic sequences of TEM images, often referred to as "movies." Dragonfly, a deep learning framework, enhances the analysis of these movies by automating various tasks and Key Steps in TEM Movie Processing Analysis with Dragonfly Deep Learning in the following procedures:

1. Data Preparation:

The first step involves preparing the TEM movie data for analysis. This includes converting the movie frames into a suitable format, ensuring proper alignment, and potentially applying pre-processing steps such as noise reduction or contrast enhancement.

1. Training Deep Learning Models:

Dragonfly deep learning involves training neural network models to recognize and classify specific features or objects of interest within the TEM movie frames. This training process requires annotated data where these features are manually labeled. For example, if the goal is to track nanoparticles or structural changes in the movie, the neural network is trained to identify and locate them accurately.

1. Feature Extraction:
2. Object Tracking:
3. Segmentation and Masking:

The deep learning model can segment specific regions of interest within the TEM movie frames. This enables more precise analysis by focusing on specific areas or objects, reducing noise and improving accuracy.

1. Quantitative Analysis:

Dragonfly can perform quantitative measurements on the extracted features, such as particle size distribution, velocity, intensity changes, and more. These measurements provide valuable insights into the underlying phenomena being studied.

**Supplementary Notes:**

**Supplementary Note 1: electron beam effect**

The electron beam effect on in situ TEM imaging of ZIF-67 growth dynamics involves considerations of beam-induced heating, radiation damage, and sample drift^1-3^. Control of beam dose, imaging conditions, and sample preparation is crucial to minimize artifacts and accurately capture real-time growth processes^4, 5^. Understanding electron-beam interactions enables insight into nucleation, growth kinetics, and structural transformations in ZIF-67, advancing our comprehension of its synthesis mechanisms and potential applications. Here, what we did is shown in the followings:

1. Reduced Beam Dose: Use a lower electron beam dose to minimize sample heating and radiation damage while still maintaining adequate imaging quality (the incident electron flux of <0.2 e^-1^ Å^–2^s^-1^).
2. Optimized Imaging Conditions: Adjust imaging parameters accelerating voltage (120 kV), and exposure time to optimize imaging conditions for reduced electron beam effects.
3. Beam Blanker: Utilize a beam blanker to intermittently switch off the electron beam during imaging intervals, reducing cumulative beam exposure and minimizing radiation damage.
4. Ex situ experiment: we do the control experiments, ex situ synthesis. A similar trend was observed by trapping the intermediates.
5. Cryo-TEM: a cryo-TEM experiment has been conducted to trap the intermediates showing non-classical nucleation, which is consistent with in situ experiments observation.

**Supplementary Note 2: ZIF-67 growth dynamics with size control**

In the growth dynamics of ZIF-67, two distinct patterns emerge: classical nucleation leading to the development of individual polyhedral particles, and non-classical nucleation resulting in the formation of aggregates that subsequently evolve into polyhedral structures. Classical nucleation follows the conventional pathway of homogeneous nucleation, where individual nuclei form and grow independently into well-defined polyhedral shapes^6, 7^. In contrast, non-classical nucleation involves the initial aggregation of precursor species, followed by their coalescence and rearrangement into larger polyhedral structures. This phenomenon is consistent with theories of non-classical nucleation, which suggest that pre-existing clusters or aggregates can serve as templates for further growth, resulting in the formation of complex structures through particle aggregation and coalescence^5, 8, 9^. The observation of both classical and non-classical nucleation pathways in ZIF-67 growth dynamics underscores the importance of considering multiple mechanisms in understanding the formation of MOF particles.

**Supplementary Note 3: sintering method effect on morphology**

Ultrafast high-temperature sintering (UHS) of ZIF-67 can yield nanocages with improved morphology and relatively high levels of nitrogen doping, especially at 1000°C for 1 minute. In contrast, traditional tube furnace sintering, due to prolonged sintering times and uneven mass and heat transfer, results in varying degrees of structural damage to ZIF-67 nanoparticles. Higher temperatures exacerbate this damage, while lower temperatures yield ZIF-67 materials with lower degrees of nitrogen doping and poorer conductivity. The following reasons need to be considered:

1. UHS: This rapid sintering process at very high temperatures within seconds promotes the formation of well-defined nanocages with improved morphology^10, 11^. The short duration prevents excessive grain growth and preserves the structural integrity of ZIF-67.
2. Traditional tube furnace sintering: Longer sintering times in conventional methods lead to prolonged exposure to elevated temperatures, resulting in uneven heating and mass transfer^12-14^. This can cause structural defects and degradation in ZIF-67 nanoparticles, particularly at higher temperatures where diffusion processes are accelerated.
3. Effect on N-doping: UHS sintering of ZIF-67 results in a high level of nitrogen doping, whereas traditional tube furnace sintering does not, primarily due to the difference in sintering kinetics and the associated impact on nitrogen incorporation. Ultrafast sintering at high temperatures enables rapid densification and nitrogen diffusion, promoting effective nitrogen doping throughout the material^15^. In contrast, traditional tube furnace sintering involves slower heating and longer dwell times, limiting the nitrogen diffusion kinetics and leading to lower nitrogen doping levels in the final products.
4. Effect on Co nanoparticle size: In ultrafast UHS sintering, the high temperatures reached in a short period induce rapid particle growth and coalescence, resulting in smaller Co nanoparticle sizes in the range of several nanometers. The rapid kinetics of sintering facilitate the formation of smaller nanoparticles before they have a chance to grow significantly^16, 17^. Conversely, traditional sintering in a tube furnace involves slower heating rates and longer sintering times. This prolonged exposure to elevated temperatures allows for greater particle growth and agglomeration, leading to larger Co nanoparticle sizes, typically around 20 nm.

**Supplementary Note 4: Li-S battery performance analysis**

The enhanced stability and high capacity of Li-S batteries using UHS sintering of ZIF-67 carbon as the cathode compared to traditional sintering can be attributed to several key factors, including the unique properties of the carbon material synthesized through ultrafast sintering:

1. Nanocage and Hollow Structure: During UHS sintering, ZIF-67 undergoes rapid heating and sintering, resulting in the formation of nanocage and hollow structures within the carbon material. These structures provide a high surface area and void space, which serve as effective reservoirs for sulfur species during the discharge and charge cycles of the battery^18, 19^. This helps to alleviate the notorious polysulfide shuttling effect, a major cause of capacity fade and reduced stability in Li-S batteries.
2. Nitrogen Doping: UHS sintering of ZIF-67 promotes high levels of nitrogen doping within the carbon matrix. The nitrogen-doped carbon serves as an efficient catalyst for the redox reactions involved in the conversion of sulfur species during battery operation^20-22^. This improves the kinetics of sulfur conversion, leading to higher capacity and better stability of the battery.
3. Smaller Co Nanoparticle: The Co nanoparticles within the carbon matrix, synthesized through UHS sintering, exhibit smaller sizes on the nanoscale. These smaller Co nanoparticles provide active sites for the adsorption and conversion of polysulfide species, facilitating their immobilization and preventing their diffusion into the electrolyte^23, 24^. This helps to maintain the integrity of the electrode and minimize capacity decay over repeated cycles.

In summary, the superior performance of Li-S batteries using UHS sintering of ZIF-67 carbon as the cathode can be attributed to the synergistic effects of nanocage and hollow structures, high nitrogen doping, and smaller Co nanoparticle size. These factors collectively enhance the stability, capacity, and cycling performance of the battery by mitigating the polysulfide shuttle effect and improving the kinetics of sulfur conversion reactions.

**Supplementary figures:**

**
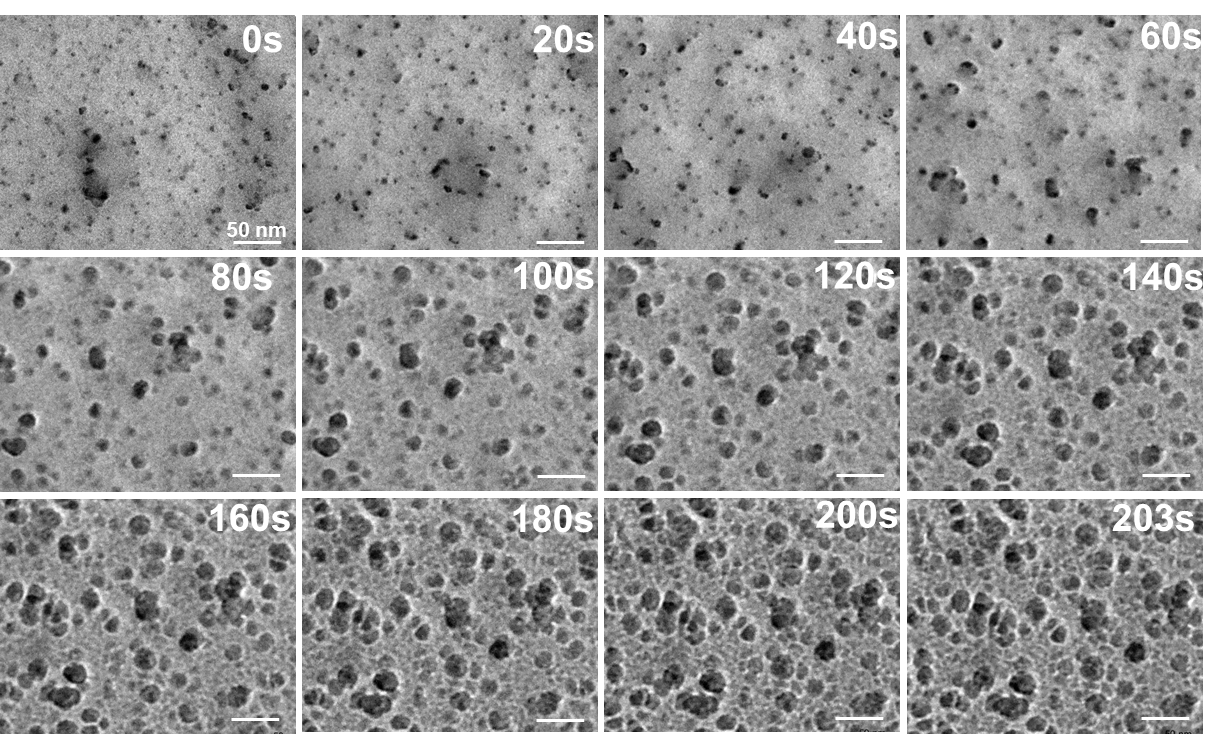
**

**Fig. S1.** Time-lapse liquid-phase TEM images capturing the nucleation process of ZIF-67 during the initial growth stage belong to classical nucleation.

**
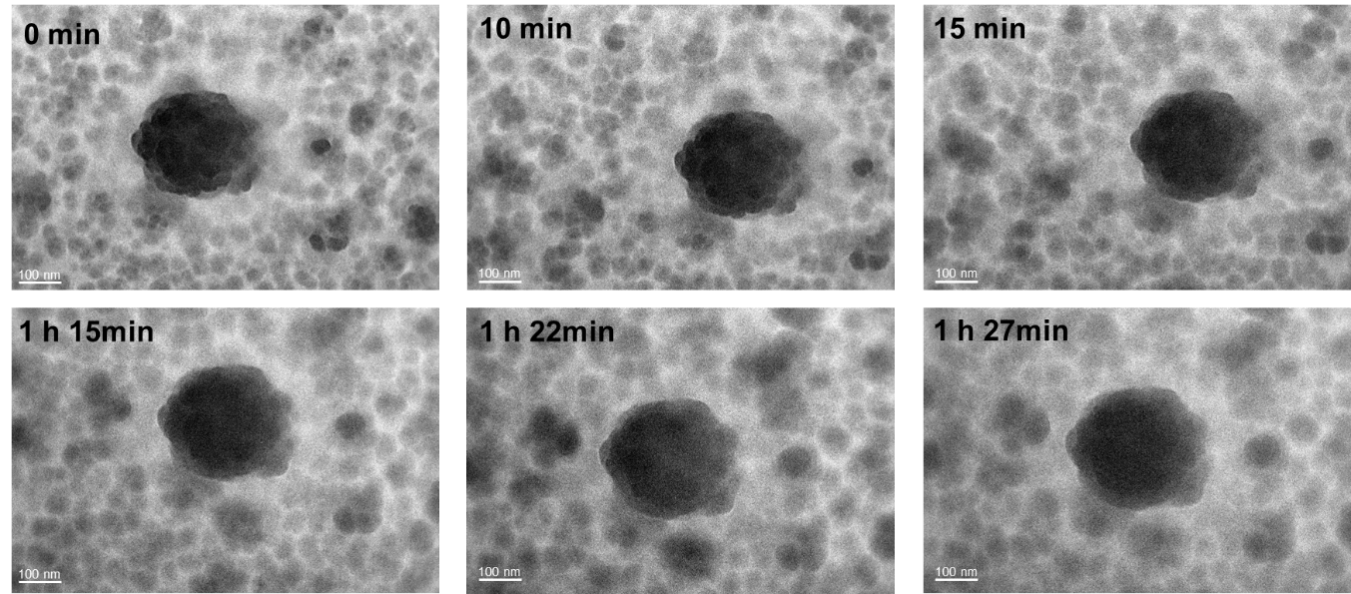
**

**Fig. S2.** Time-lapse liquid-phase TEM images capturing the process where multiple small particles of ZIF-67 aggregate to form larger particles and gradually grow into dodecahedra belong to non-classical nucleation.

**
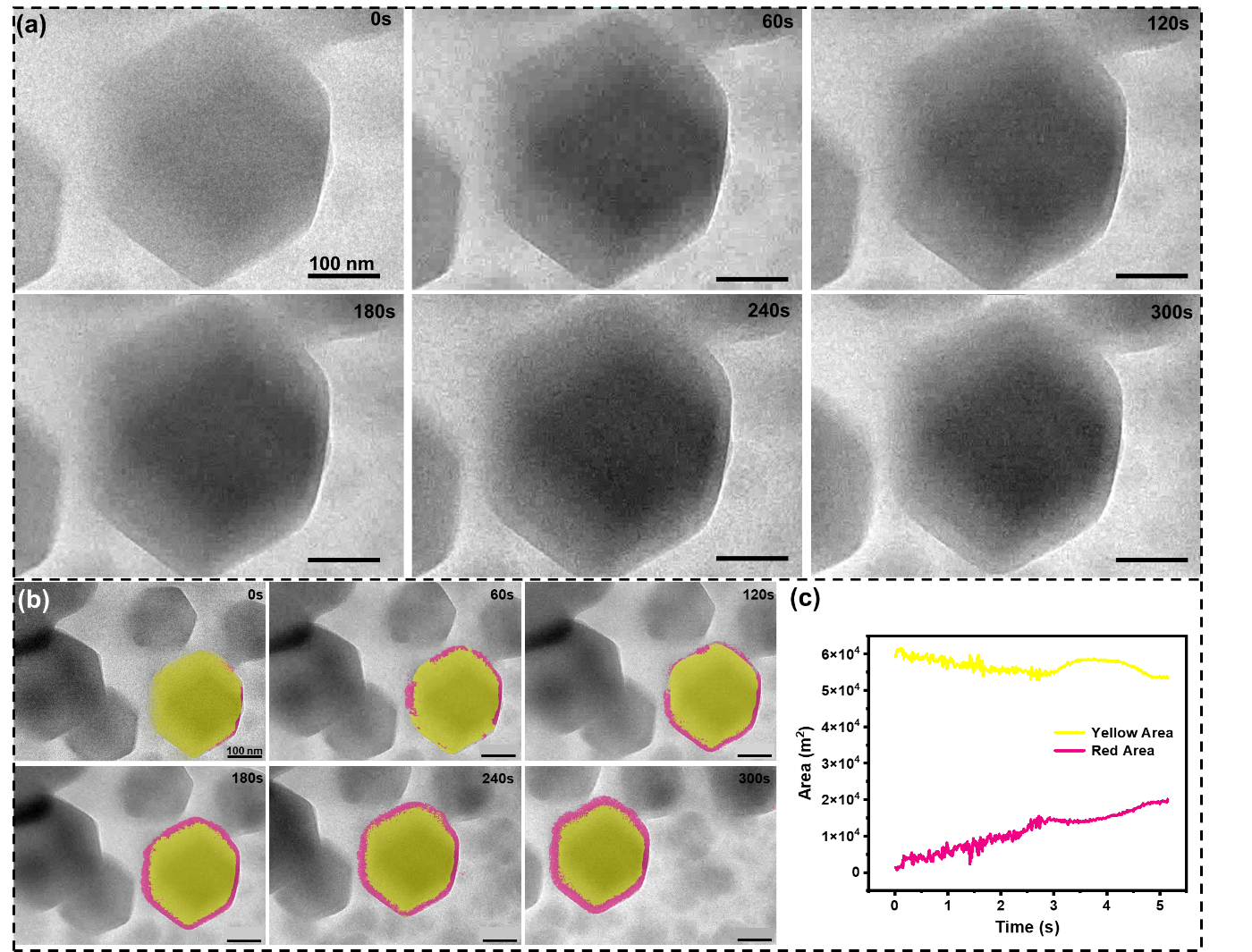
**

**Fig. S3.** (a) Time-lapse liquid-phase TEM images capturing the growth process of individual dodecahedral particles of ZIF-67, it is amorphous nanocrystal. (b) labelling the inner and outer layers of in-situ liquid-phase TEM images of ZIF-67, and (c) the curves of area change over time for the inner and outer layers via deep learning.


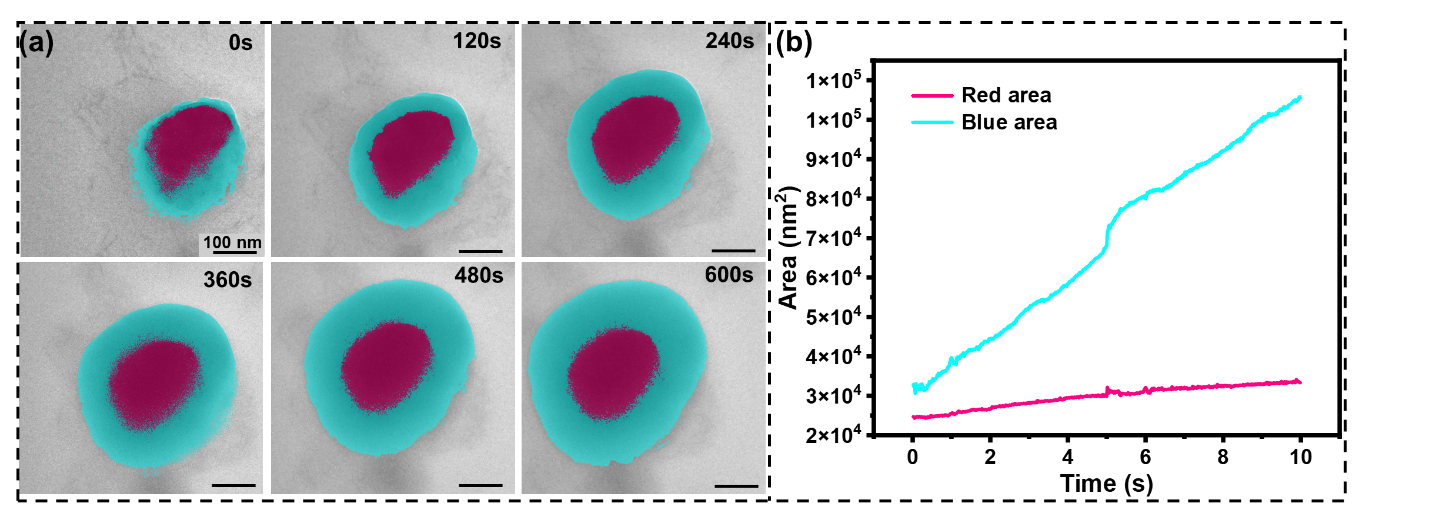


**Fig. S4.** (a) labelling the inner and outer layers of in-situ liquid-phase TEM images of ZIF-67 single particle, and (b) the curves of area change over time for the inner and outer layers via deep learning.


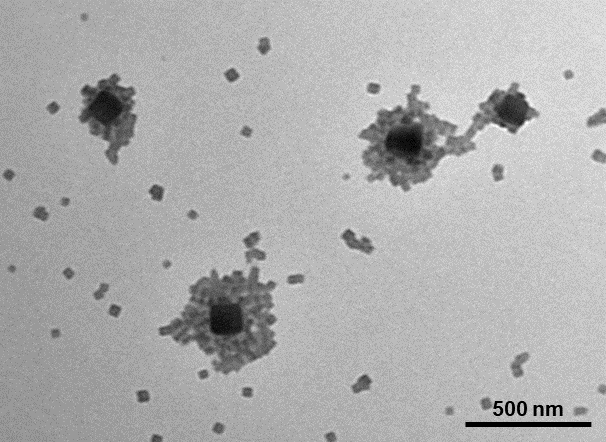


**Fig. S5.** Cryo**-**TEM image showing morphology of ZIF-67 after 3 min growth illustrate well the presence of aggregate growth dynamics (or attachment). This aligns with the observations from in-situ liquid-phase TEM experiments, a non-classical nucleation.

**Fig. S6.** Particle size distribution of ZIF-67 particles before mixing, 2 minutes growth, and 20 minutes growth measured by NanoZS90.


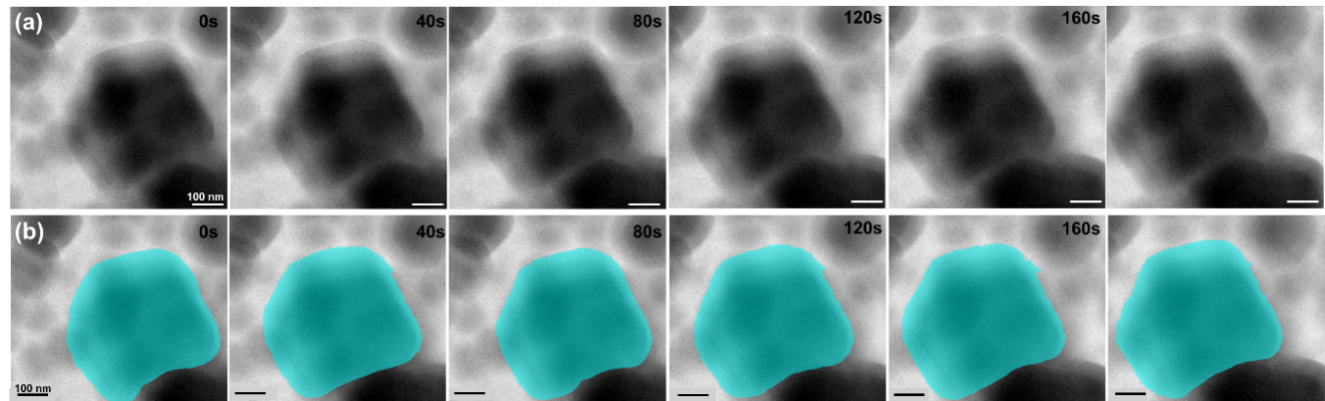


**Fig. S7.** (a)Time-lapse liquid-phase TEM images capture the process where multiple ZIF-67 spherical nanocrystals gradually aggregate and grow into cubic crystal, (b)labelling the in-situ liquid-phase TEM images.


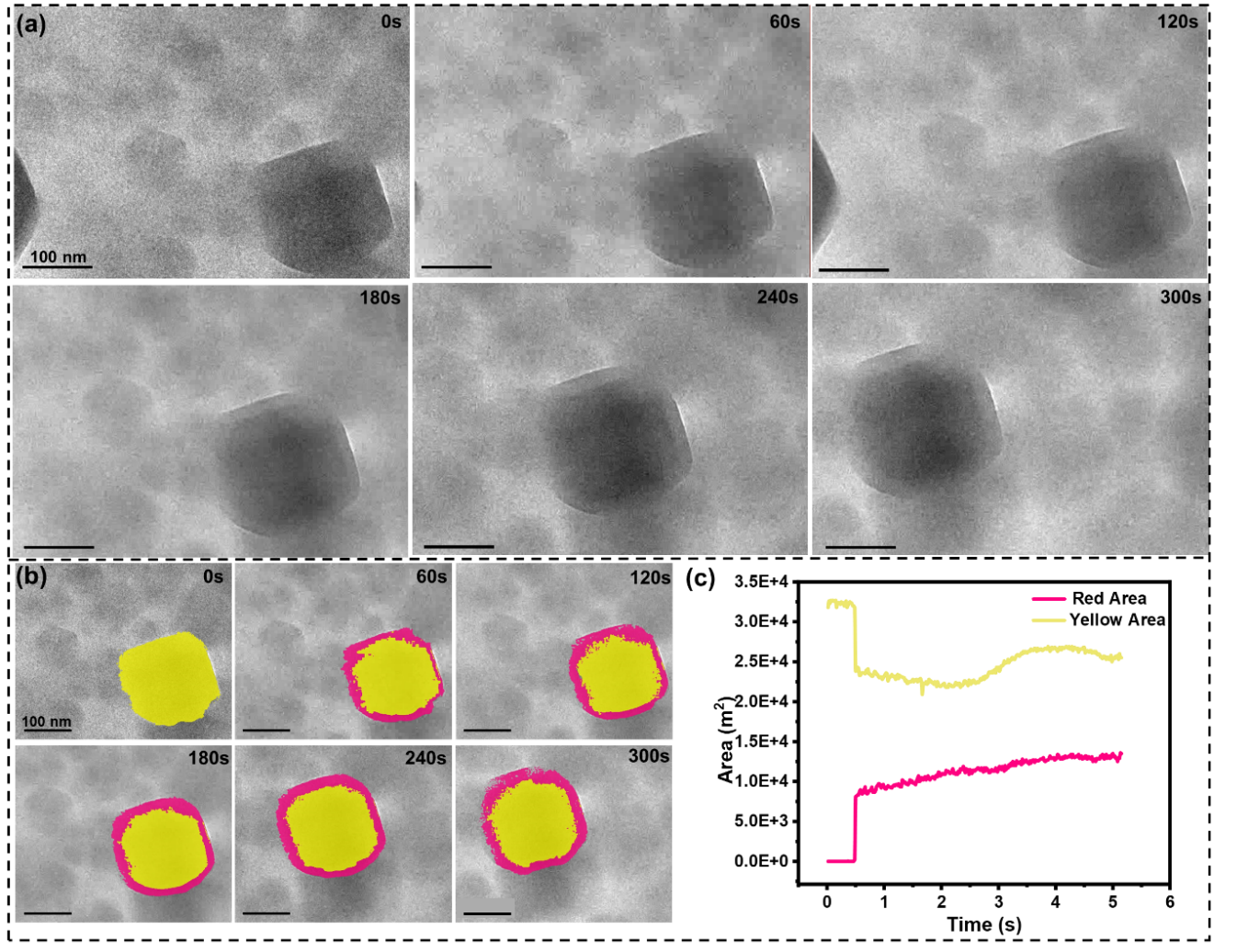


**Fig. S8.** (a) Time-lapse liquid-phase TEM images capturing the growth process of individual cubic crystal of ZIF-67, it is amorphous nanocrystal. (b) labelling the inner and outer layers of in-situ liquid-phase TEM images of ZIF-67, and (c) the curves of area change over time for the inner and outer layers calculated via deep learning.


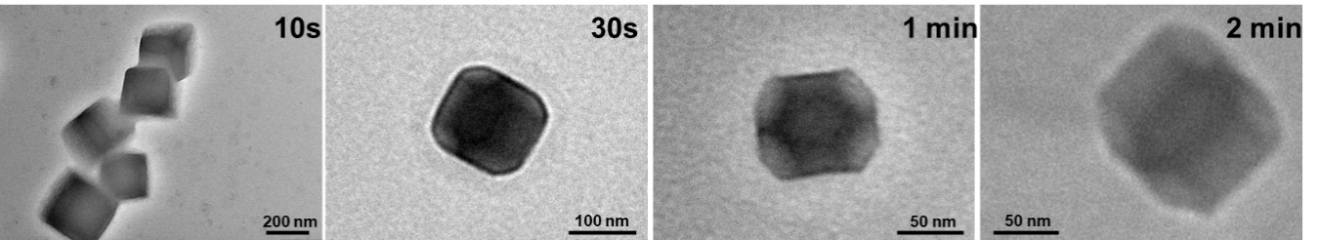


**Fig. S9.** The TEM images depict the morphology of intermediates at different growth stages of ZIF-67. Experimentally, liquid was withdrawn from the mother liquor at various time points and quenched to obtain the morphology of samples at those time points. This aligns with the observations from in-situ liquid-phase TEM experiments, a classical nucleation. Note: this is not an in situ experiment.

**Fig. S10.** The XRD spectra of ZIF-67 at different growth time.

**
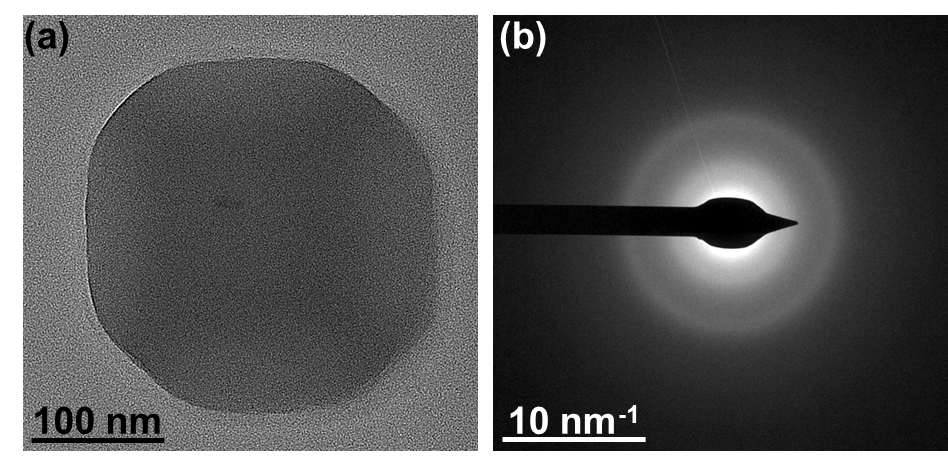
Fig. S11.** (a) TEM image and (b) selected area electron diffraction pattern (SAED) of ZIF-67 nanoparticle, ZIF-67 grown for 30 s in mother solution. It is amorphous crystal.

**Fig. S12.**XRD spectra of sintered ZIF-67 nanoparticles at different temperatures and times.


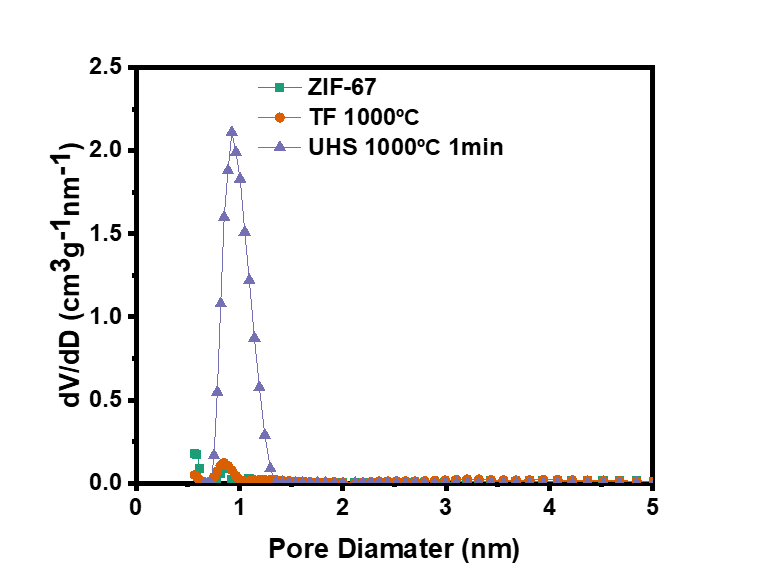


**Fig. S13.**Pore size distribution of the samples. (d) N XPS spectra of sintered ZIF-67 at 1000 °C for 10 s with UHS.


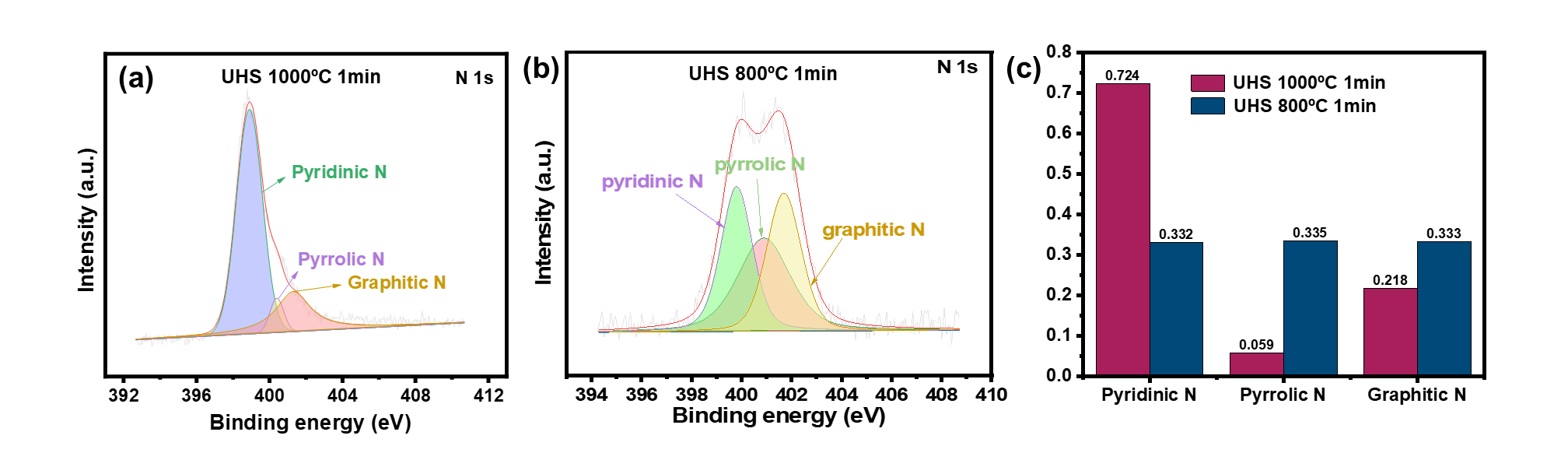


**Fig. S14.** (a) N XPS spectra of sintered ZIF-67 at 1000 °C for 1 min with UHS. (b) Graph showing different types of nitrogen content calculated from XPS spectra**.** (c) different types of N (N- bonding) element ratio in the different samples under UHS sintering treatment.


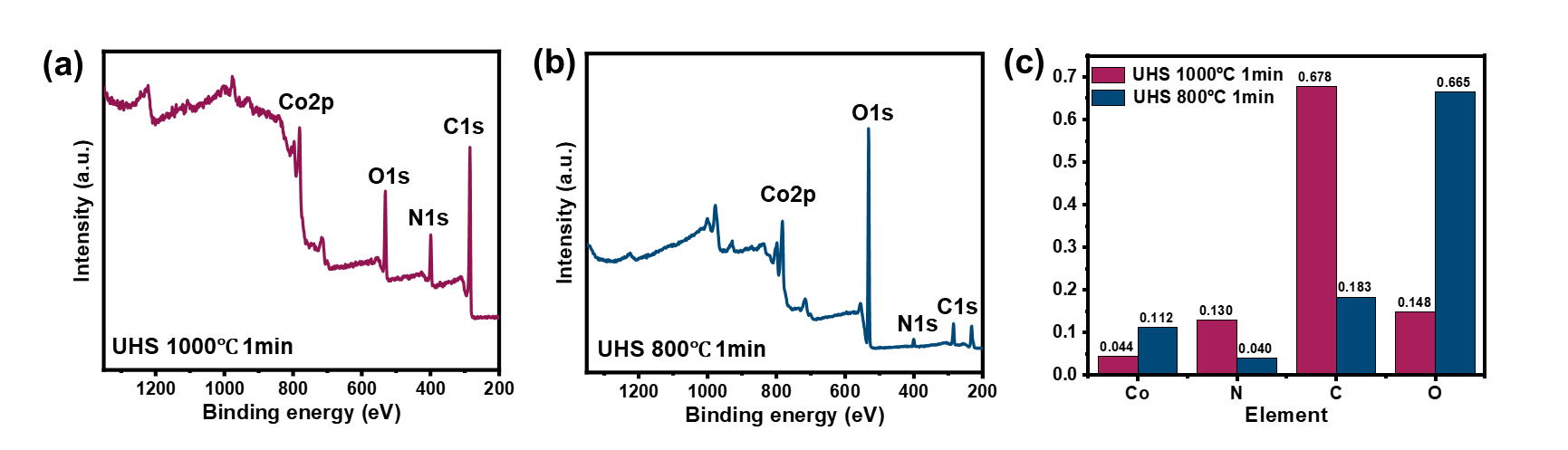


**Fig. S15.** XPS curve of ZIF-67 via UHS at (a) 800 °C, and (b)1000 °C 1 min respectively. (c) the elements ratios in the two samples.


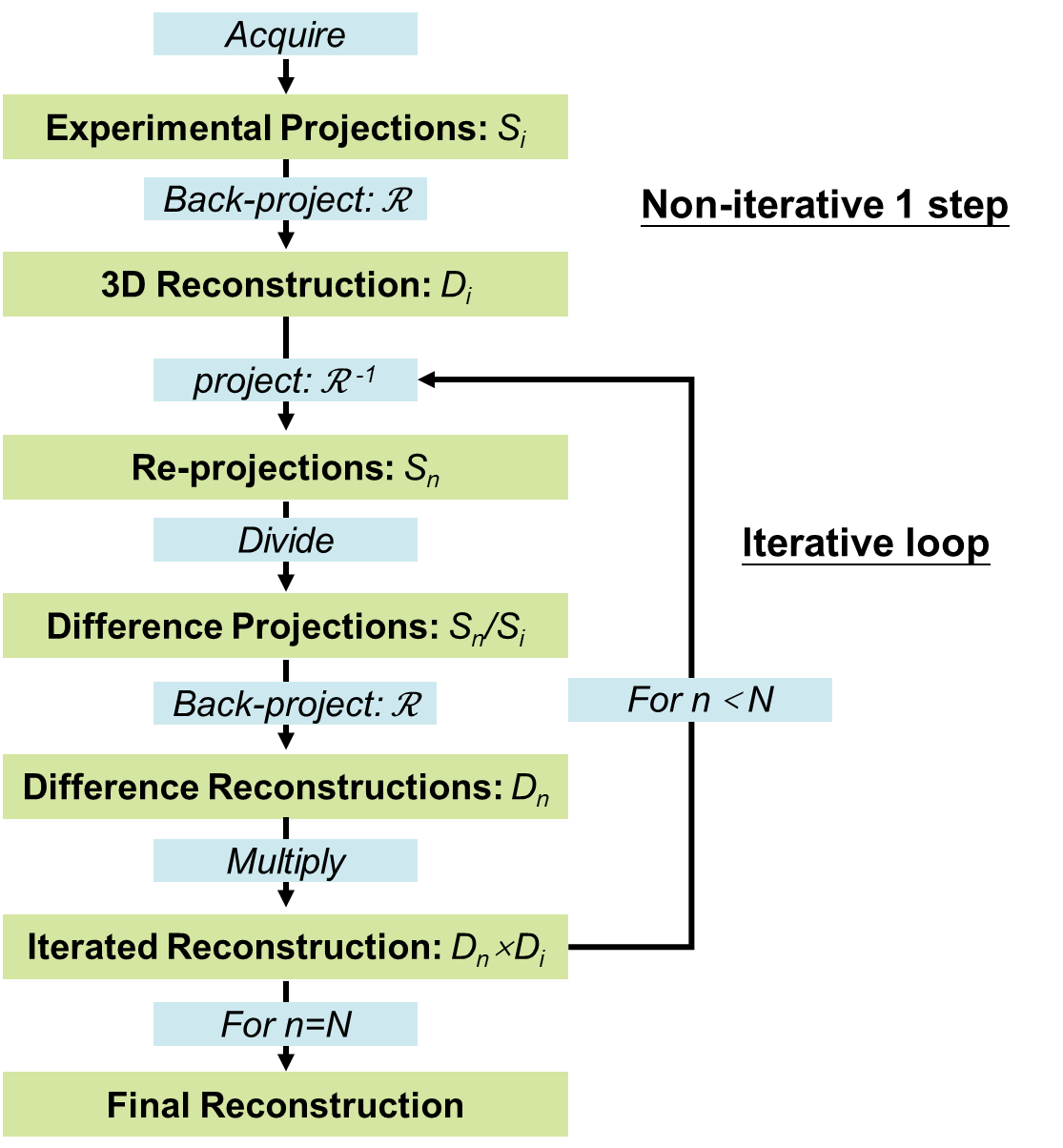


**Fig. S16.** The flowchart represents the process of 3D electron tomography.


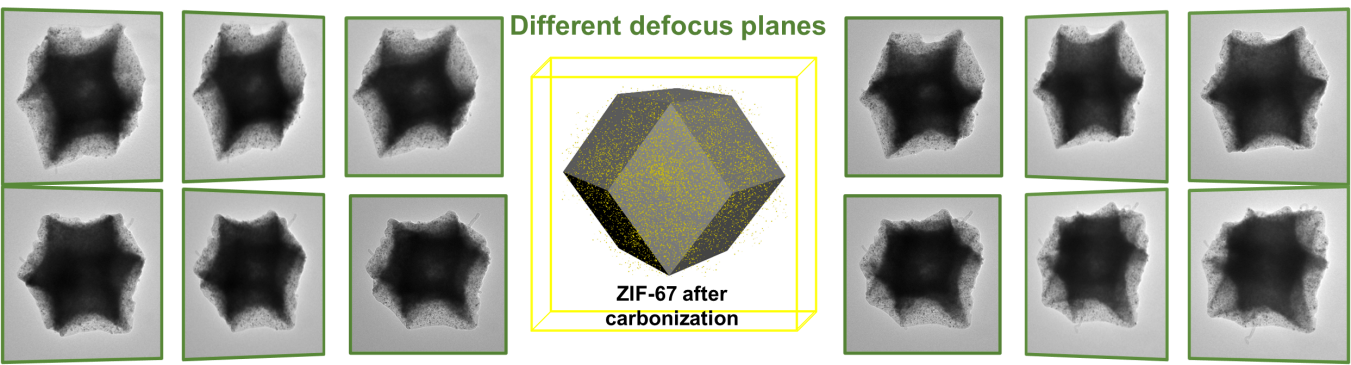


**Fig. S17.** TEM images of sintered ZIF-67 via UHS 1000 °C for 1 min collected at different defocus planes.


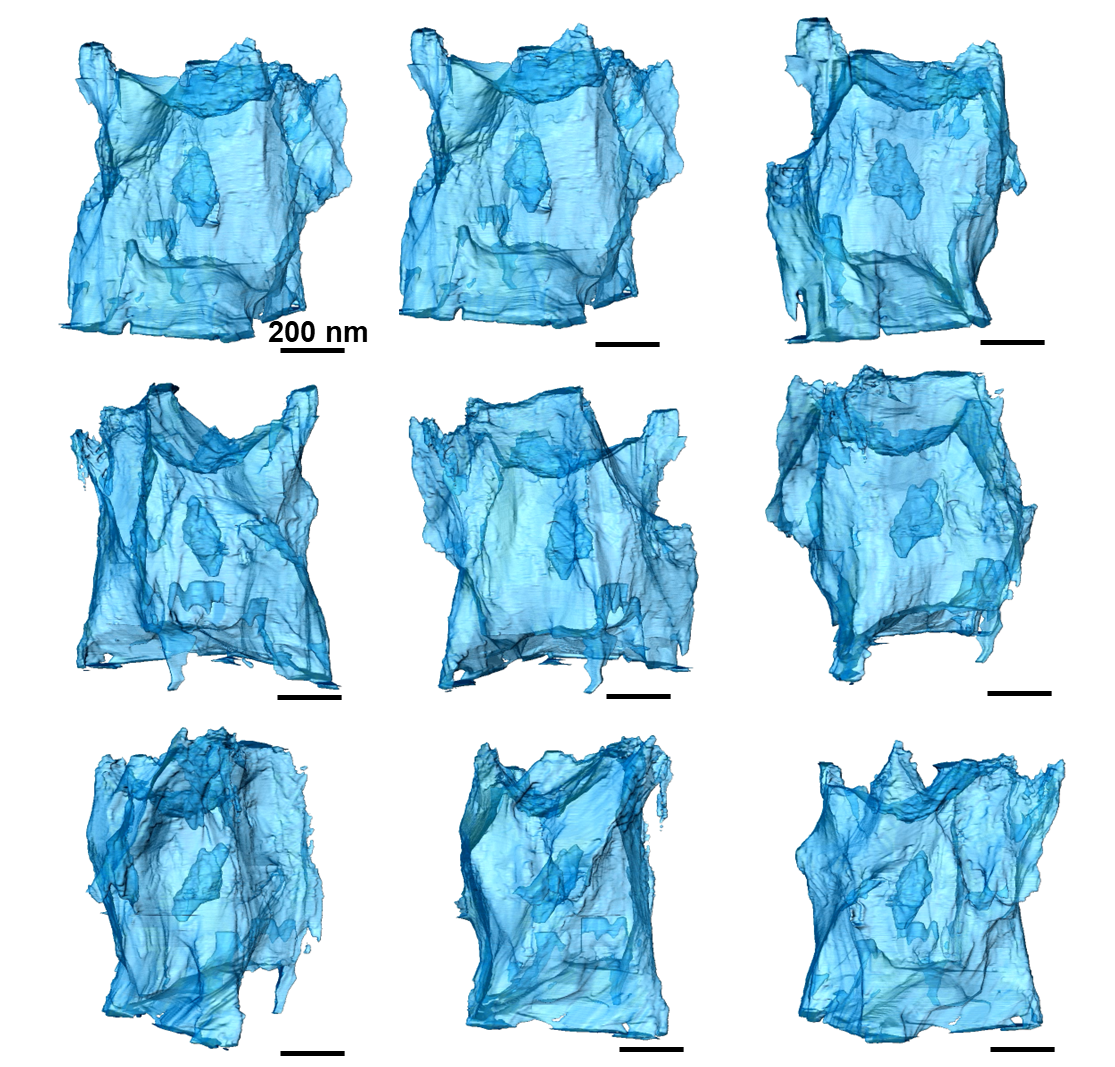


**Fig. S18.** 3D visualization of ZIF-67 after UHS sintering at 1000 °C for 1 minute, showing images from different view angles after volume rendering. It is clear to see the nanocage morphology.


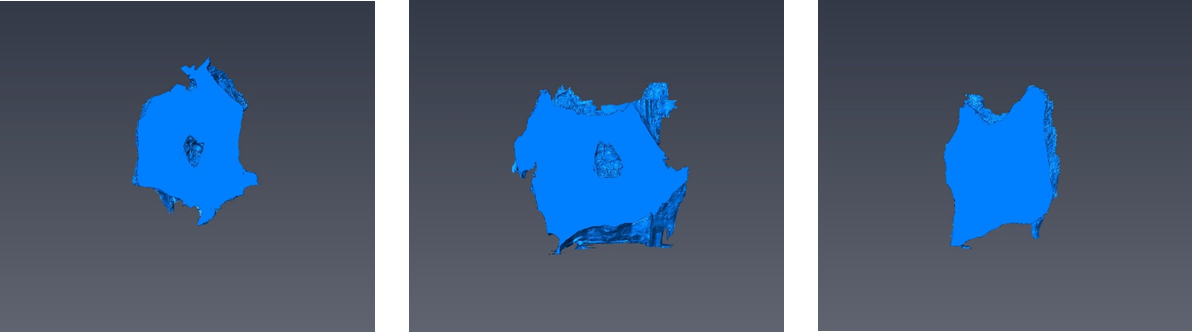


**Fig. S19.** 3D visualization of ZIF-67 after ultra-high-temperature treatment at 1000 °C for 1 minute, showing images from cross section. It is clear to see the hollow core within nanocage.


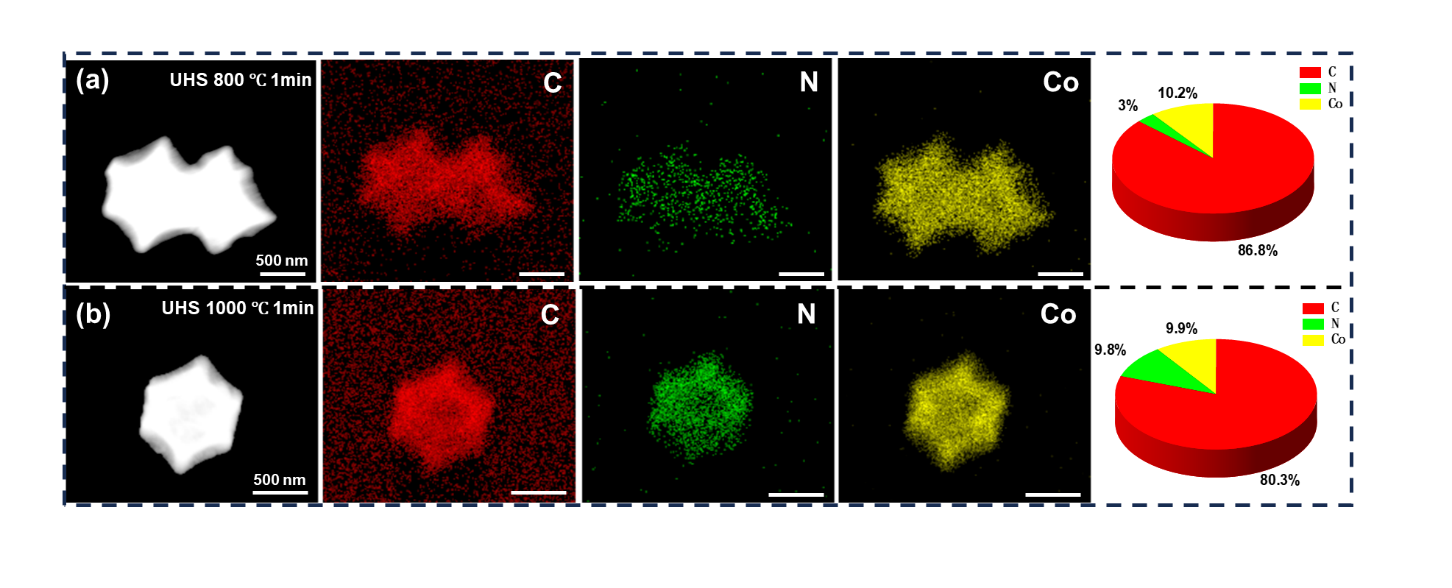


**Fig. S20.** EDX maps of the samples sintered at (a) 800 °C, and (b) 1000 °C for 1.0 min via UHS showing N and Co doped with high concentration. Left: TEM and SEM image of sintered ZIF-67, right: the relative EDX color maps of the same particle.


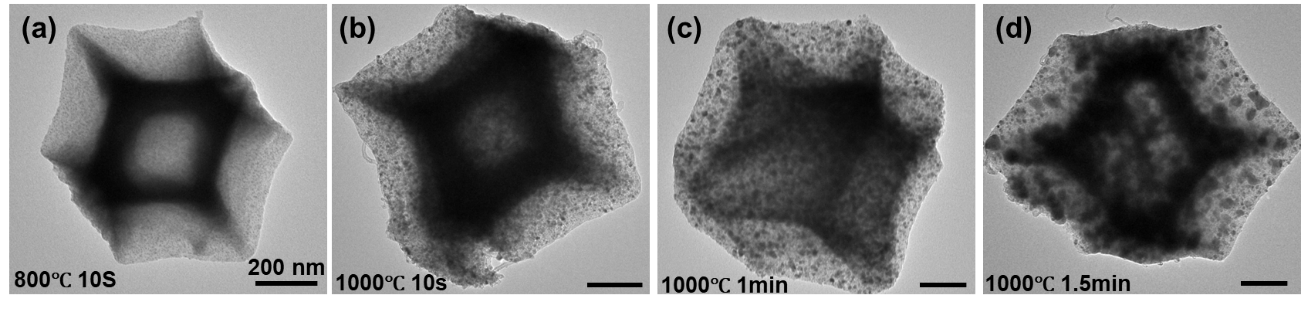


**Fig. S21.**The TEM images of ZIF-67 at different UHS calcination temperatures and times show variations in its morphology and Co NPs size.


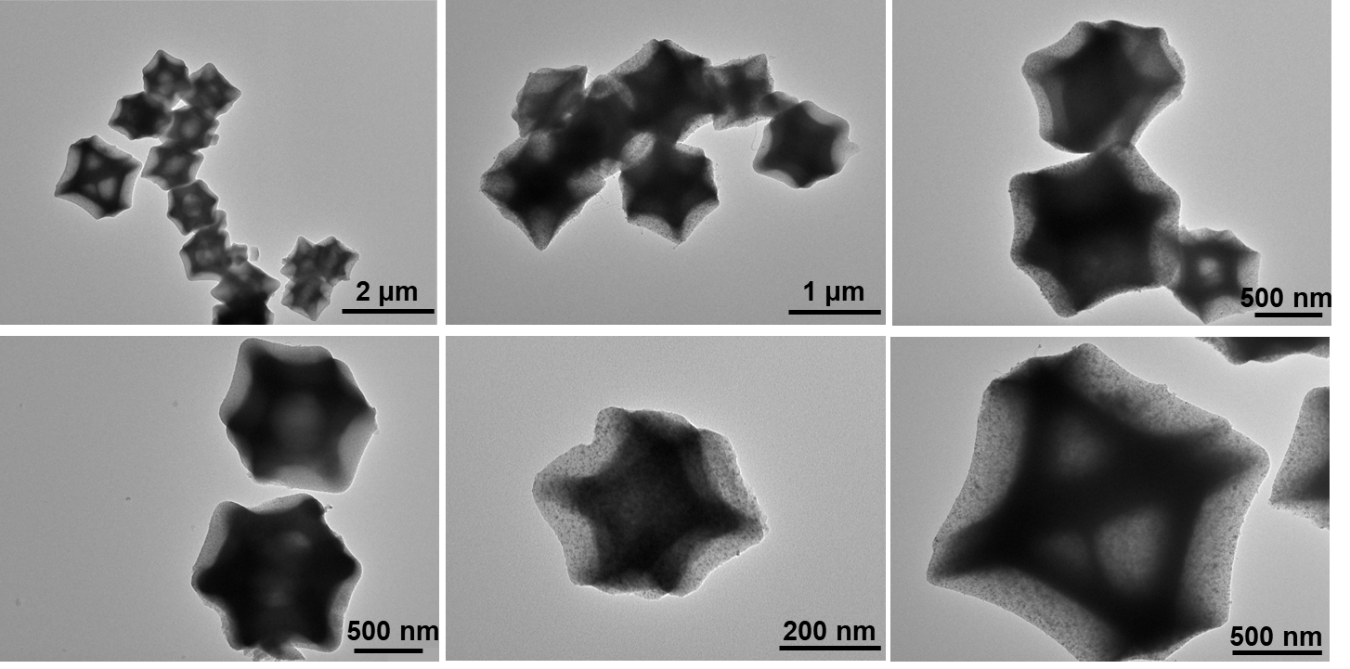


**Fig. S22.** TEM images of ZIF-67 sintered at 1000 °C for 10 seconds via UHS.


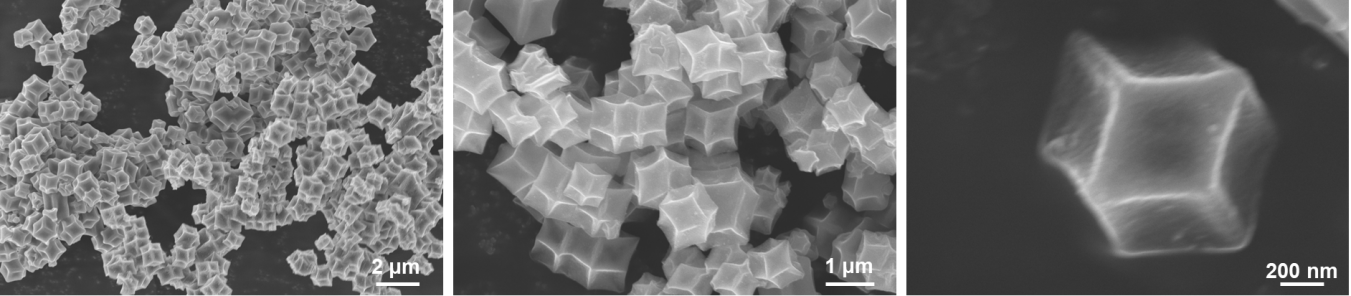


**Fig. S23.** SEM images of samples sintered at 1000 °C for 10 seconds via UHS.

**
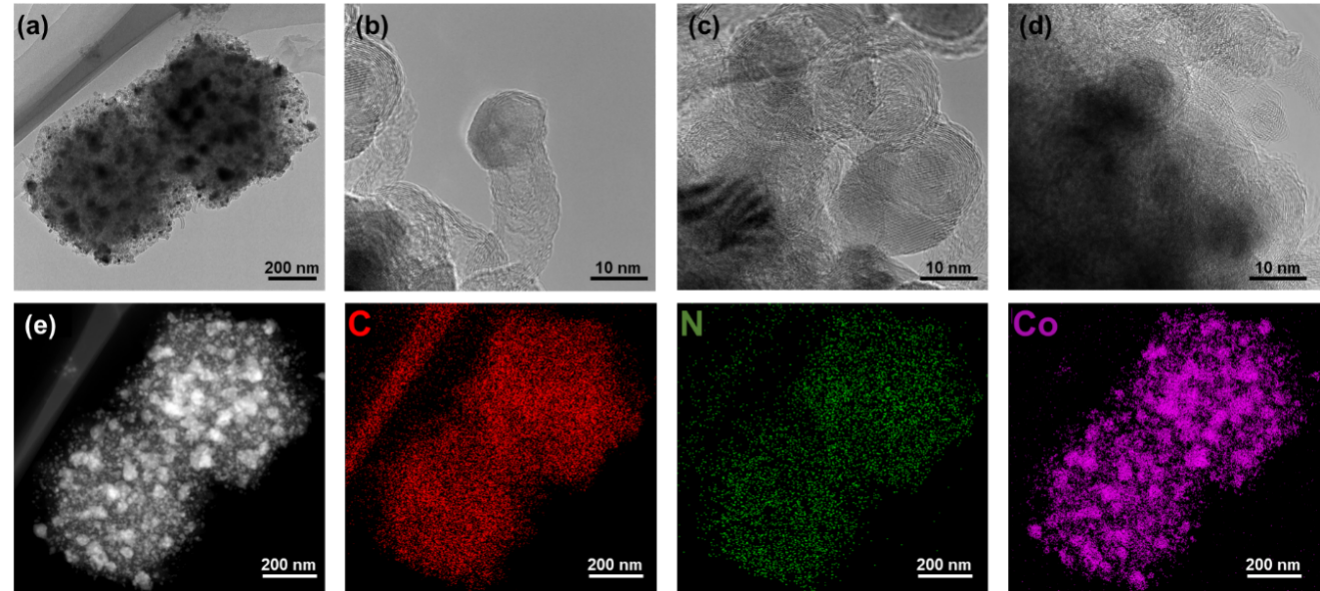
**

**Fig. S24.** (a-d) HRTEM images of samples sintered at 1000 °C for 1.5 min with UHS, (e) EDX maps of the sintered ZIF-67 showing N and Co doped with high concentration.


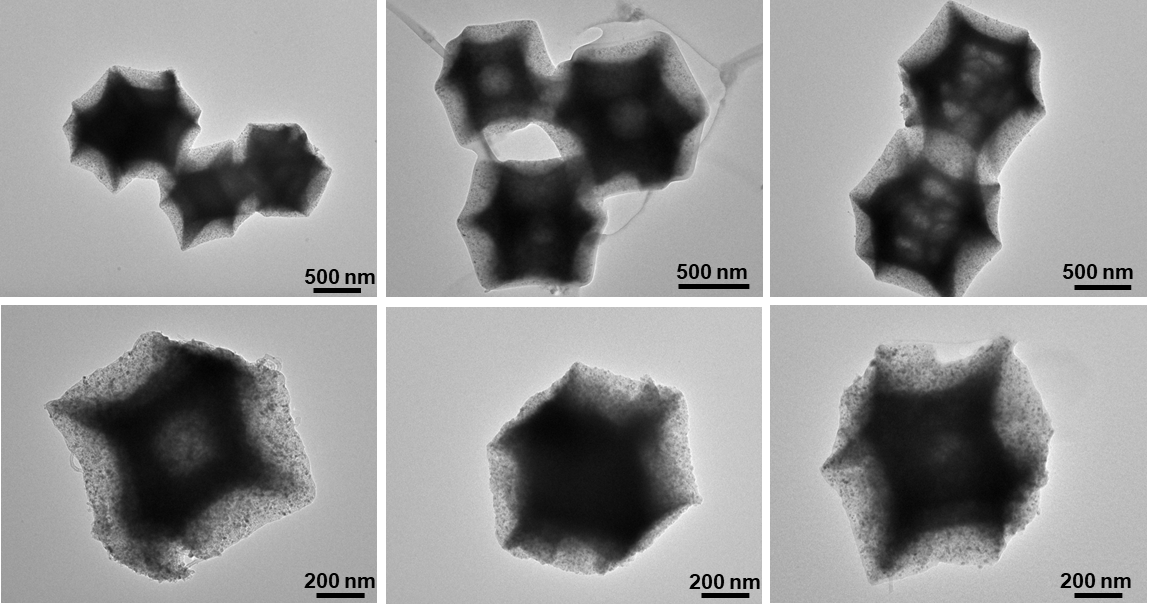


**Fig. S25.** TEM images of ZIF-67 sintered at 1000 °C for 1 min via UHS.


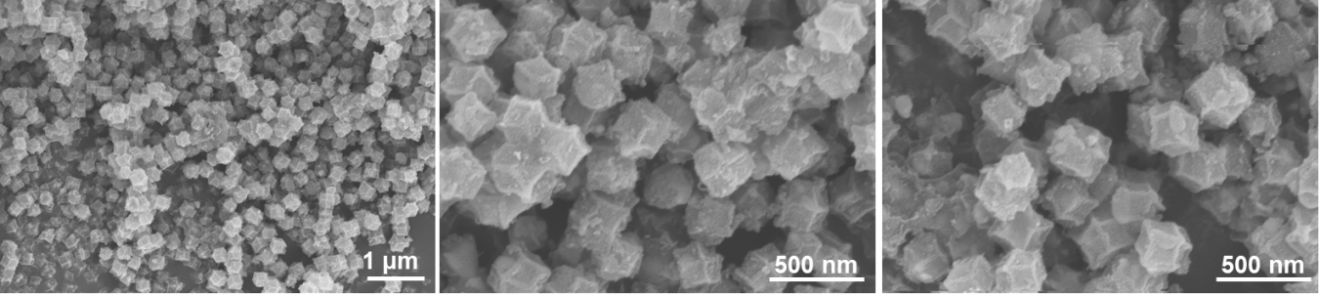


**Fig. S26.**SEM images of ZIF-67 sintered at 1000 °C for 1 min via UHS.


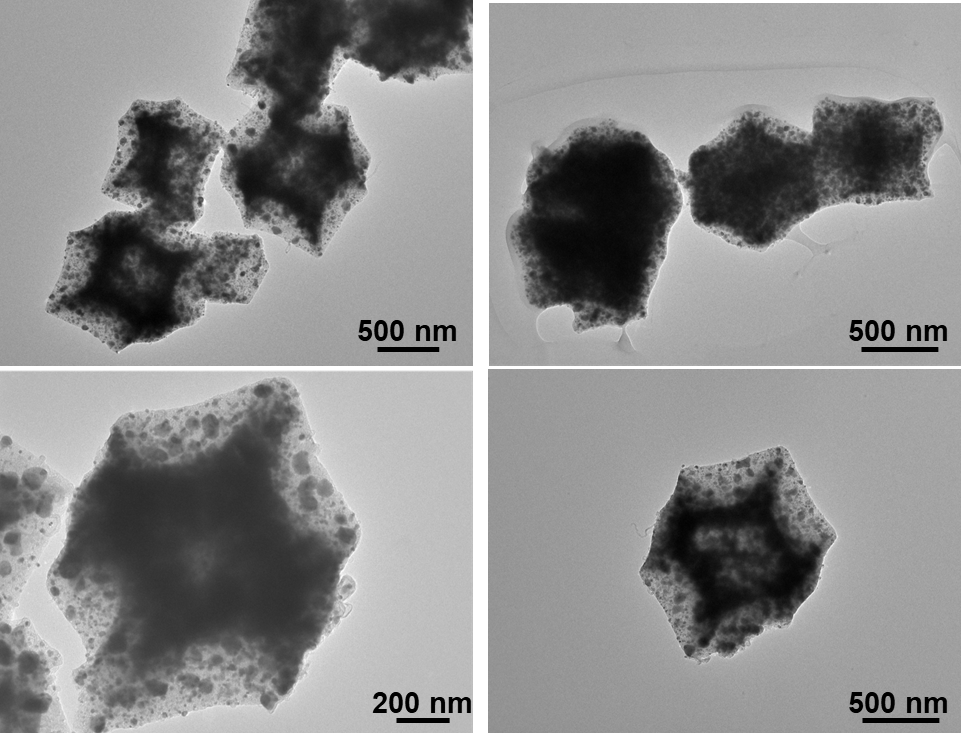


**Fig.S27.** TEM images of samples sintered at 1000 °C for 1.5 min with UHS. Note: the large Co nanoparticles have been etched before it was applied in Li-S battery. However, the electrochemical performance is not good.


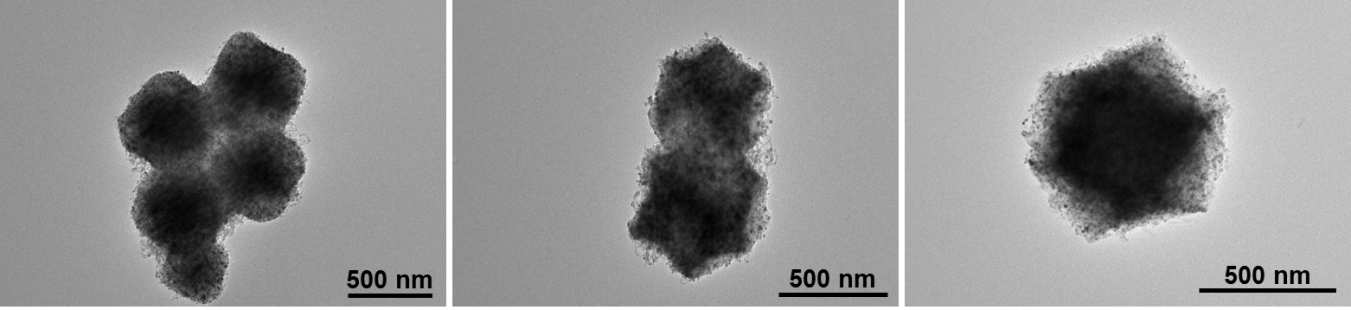


**Fig. S28.** TEM images of ZIF-67 sintered at 650 °C for 1 min via UHS.

**
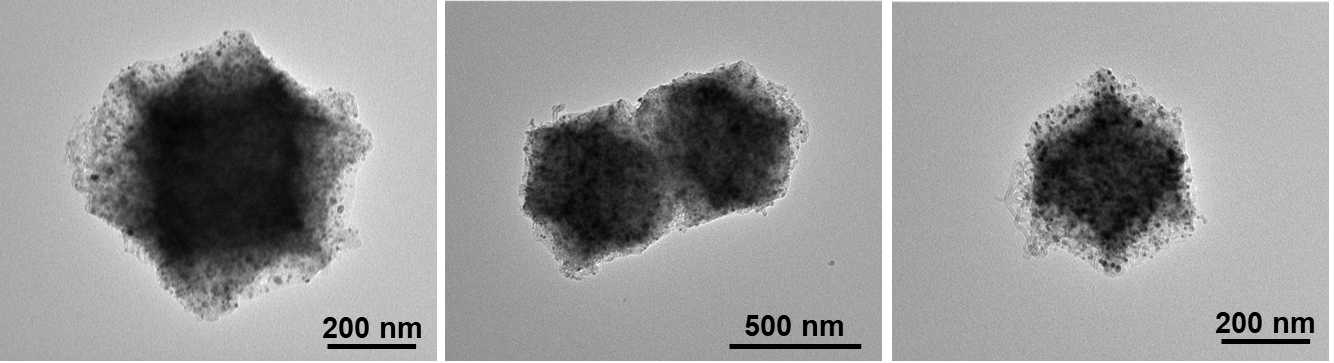
**

**Fig. S29.** TEM images of ZIF-67 sintered at 800 °C for 1 min via UHS.


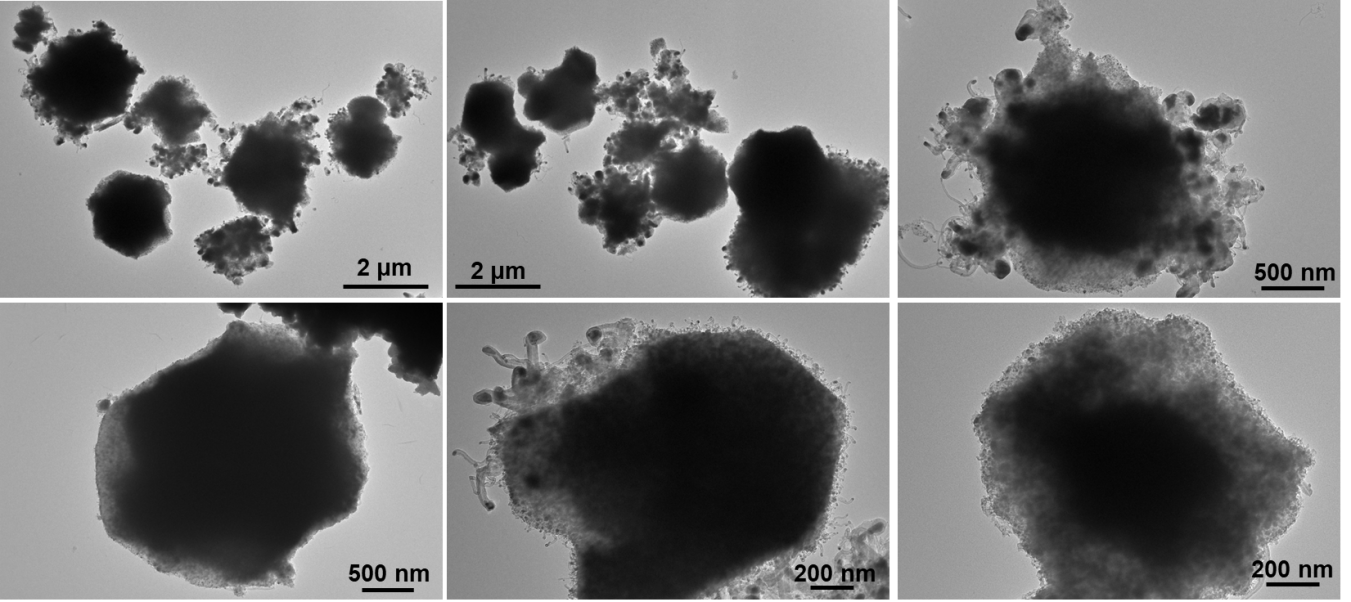


**Fig. S30.** TEM images of ZIF-67 after tube furnace carbonization at 650 °C for 2 hours. (Conventional sintering)


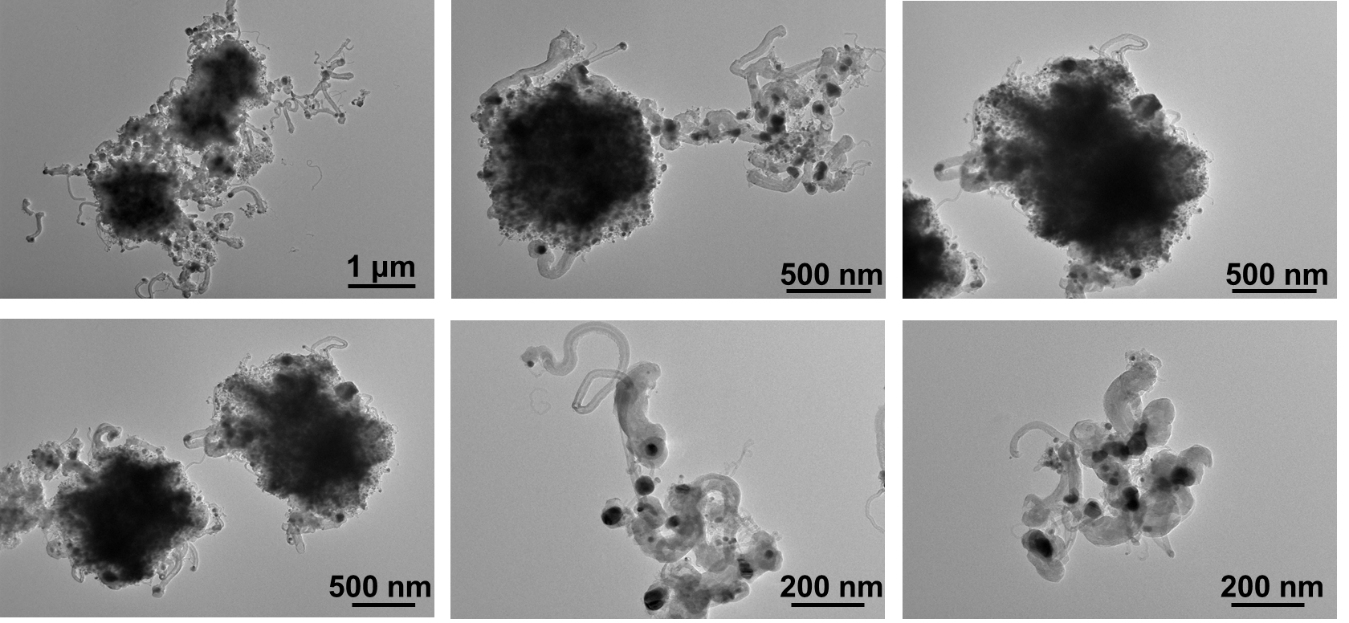


**Fig. S31.** TEM images of ZIF-67 after tube furnace carbonization at 800 °C for 2 hours. (Conventional sintering)


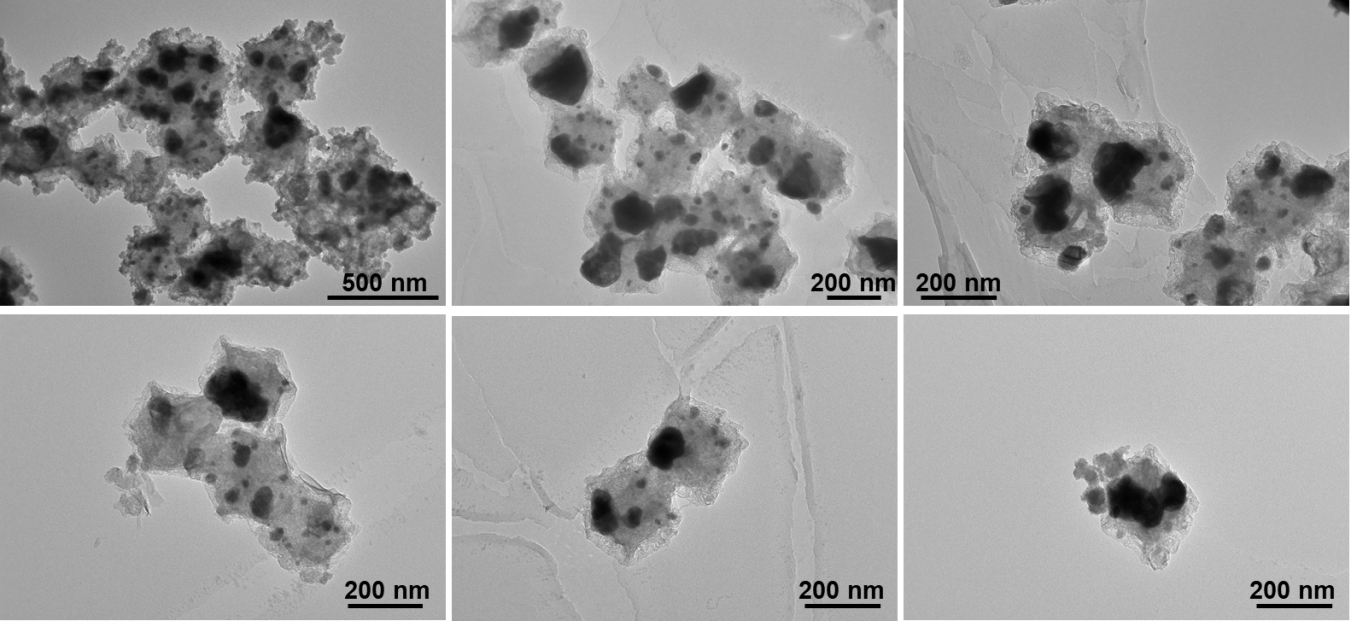


**Fig. S32.** TEM images of ZIF-67 after tube furnace carbonization at 1000 °C for 2 hours. (Conventional sintering)

**Fig. S33.** TGA curves of UHS 1000 °C 1 min、UHS 650 °C 1 min、UHS 800 °C 20 s、UHS 800 °C 1 min and TF 800 °C to show Sulphur loading dosage in different samples.

**
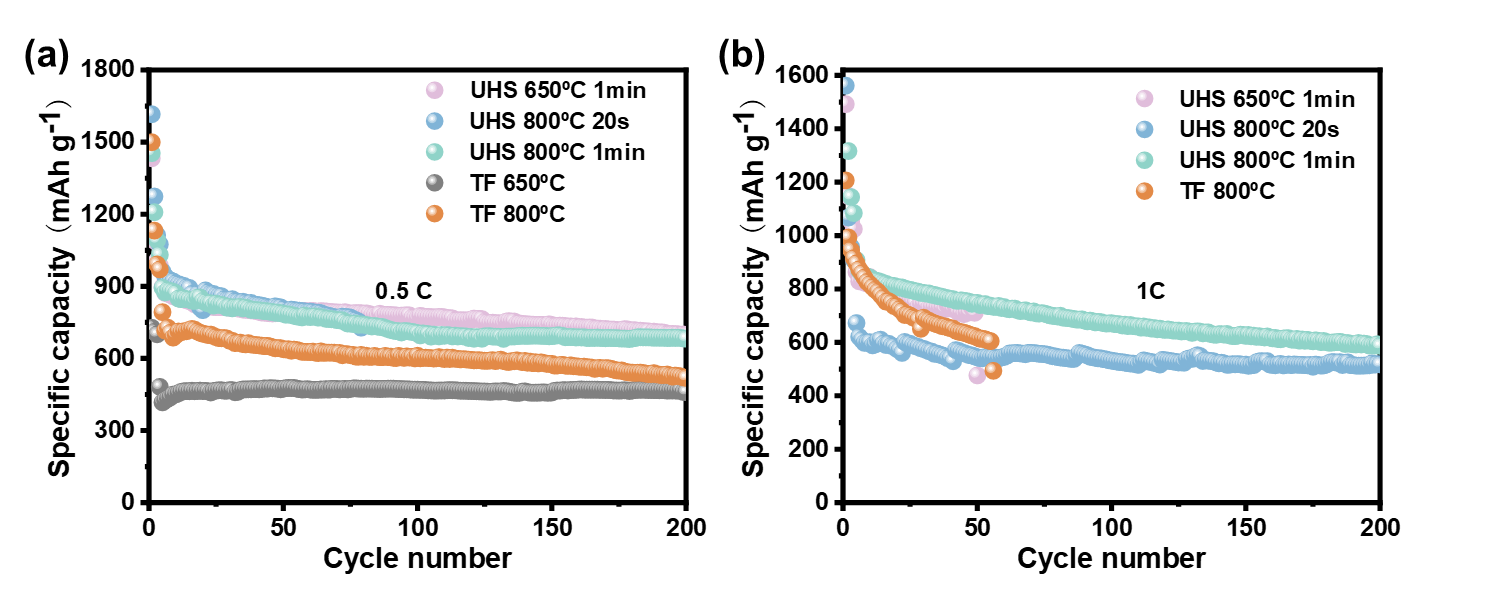
**

**Fig. S34.** (a) Cycling curves of ZIF-67 sintered via UHS 650 °C for 1 minute, UHS 800 °C for 20 seconds, UHS 800 °C for 1 minute, TF 650 °C, and TF 800 °C at 0.5 C respectively, (b) Cycling curves of ZIF-67 sintered via UHS 650 °C for 1 minute, UHS 800 °C for 20 seconds, UHS 800 °C for 1 minute and TF 800 °C at 1 C respectively. The above results indicate that samples synthesized via tube furnace sintering or UHS 800 °C sintering exhibit poor electrochemical performance.

**
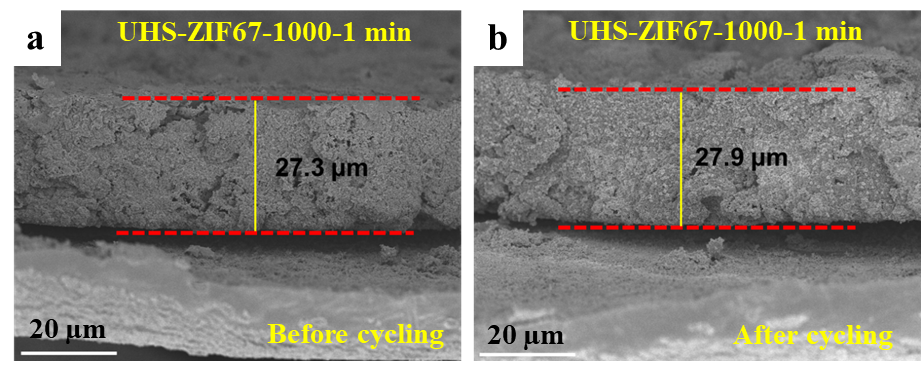
**

**Fig. S35.** (a and b) SEM images show the cross-sectional of the electrode sample before and after cycling respectively whose cathode is composed of ZIF-67 via UHS 1000°C-1-minute.

Note: According to our experimental observations, during the preparation of SEM samples, the cathode materials are prone to delamination and detachment due to external forces. The actual samples exhibit smoother and denser cross-sectional morphologies without treatment. Therefore, these images are for reference only, indicating that the samples are relatively stable and capable of supporting long-term battery cycling.


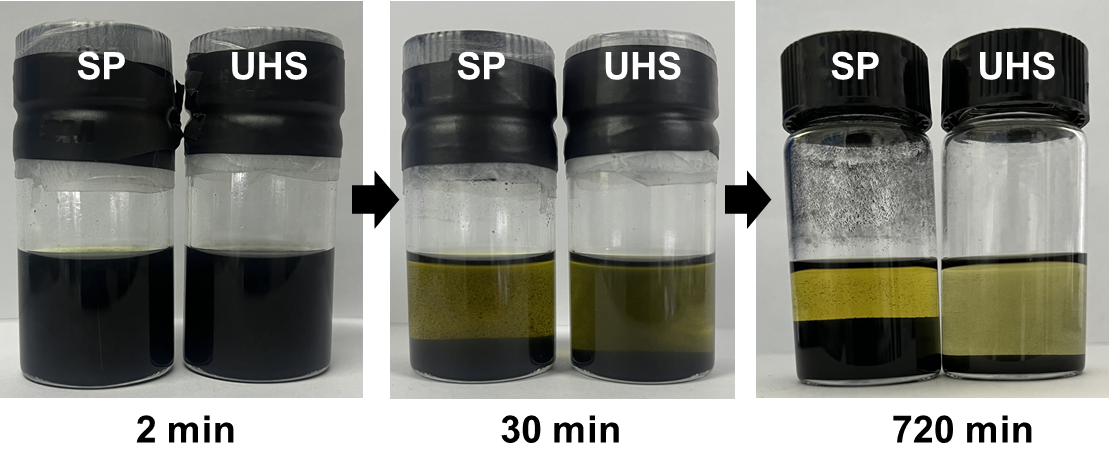


**Fig. S36.** Optical photos of visualized adsorption experiment: different materials was assessed by dispersing SP or UHS-ZIF- 1000°C-1-minute into a 5 mmol L^–1^ Li_2_S_6_ solution and allowed to stand for up to 12 h.

**Supplementary Tables**

**Table S1.** Morphology comparison with literatures

| Material | Morphology | Ref. |
| --- | --- | --- |
| ZnS@Co-N-C | Hollow core-shell composite | 25 |
| S/CoS_2_-NC | Synthesis of S/CoS_2_ Nanoparticles-Embedded N-doped Carbon Polyhedrons from Polyhedrons ZIF-67 | 26 |
| Co-NC@V_2_O_3_-sp | VO hollow sphere structure of Co-NC@VO-sp | 27 |
| Co-PB@S | Cherry blossom derived micron carbon sheets loaded with Co based particles | 28 |
| UHS 1000 °C 1 min | Hollow carbon nanocage with Zn-N-doping | This work |

**Note:** Our method is simple, short time, and low energy consumption.

**Table S2.** The comparison of electrochemical performance with similar materials in literature

| Ref. | Cathode  material | Current  density | Cycles | Capacity  retention  (mA h g^-1^) |
| --- | --- | --- | --- | --- |
| 29 | CNT@TiO_2-x_–S | 1 C | 500 | 590 |
| 30 | HPNC-S | 0.2 C | 300 | 520 |
| 31 | GSH@APC-S | 1 C | 150 | 877 |
| 32 | Fe/Co-N-HPC | 0.2 C | 80 | 1015 |
| 33 | S/YS NiO@HCSs | 1 C | 300 | 568 |
| 34 | S@Sr_8_Ti_7_S_21_ | 1C | 400 | 520 |
| **This work** | UHS-ZIF67-1000 °C-1 min | 1 C | 200 | 718 |

**Table S3.** The data of Co nanoparticles size change due to different sintering temperatures

| **Sample Name** | **Particle Size (nm)** |
| --- | --- |
| UHS 1000°C 10s | 4-6 |
| UHS 1000°C 1min | 5-6 |
| UHS 1000°C 1.5min | 21-25 |
| TF 800°C | 30-35 |
| TF 1000°C | 25-50 |

**3. Supporting movies**

**Supporting movie 1**: In-situ transmission electron microscope imaging shows that the initial classical nucleation of ZIF-67 in the initial stage of growth.

Imaging conditions: The in-situ transmission electron microscope study was conducted in a 120kV TEM-1400 flash TEM (JEOL Co., Ltd., Tokyo, Japan) equipped with Gatan camera (Gatan Company, Pleasanton, California, USA). In-situ image series were obtained at the rate of 10 frames per second and the incident electron flux of <0.2 e^-1^ Å^–2^s^-1^.

Movie play speed: x 30 times of real speed

**Supporting movie 2**: In-situ transmission electron microscope imaging shows that the process by which small particles coalesce to form large particles and gradually grow into a dodecahedron, a non-classical nucleation.

Imaging conditions: The in-situ transmission electron microscope study was conducted in a 120kV TEM-1400 flash TEM (JEOL Co., Ltd., Tokyo, Japan) equipped with Gatan camera (Gatan Company, Pleasanton, California, USA). In-situ image series were obtained at the rate of 10 frames per second and the incident electron flux of <0.2 e^-1^ Å^–2^s^-1^.

Movie play speed: x 60 times of real speed.

**Supporting movie 3**: In-situ transmission electron microscope imaging shows that the growth process of a single dodecahedral particle of ZIF-67, a classical nucleation and growth mode.

Imaging conditions: The in-situ transmission electron microscope study was conducted in a 120kV TEM-1400 flash TEM (JEOL Co., Ltd., Tokyo, Japan) equipped with Gatan camera (Gatan Company, Pleasanton, California, USA). In-situ image series were obtained at the rate of 10 frames per second and the incident electron flux of <0.2 e^-1^ Å^–2^s^-1^.

Movie play speed: x 60 times of real speed

**Supporting movie 4**: In-situ transmission electron microscope imaging shows that the growth process of a single core-shell particle of ZIF-67, a classical nucleation and growth mode.

Imaging conditions: The in-situ transmission electron microscope study was conducted in a 120kV TEM-1400 flash TEM (JEOL Co., Ltd., Tokyo, Japan) equipped with Gatan camera (Gatan Company, Pleasanton, California, USA). In-situ image series were obtained at the rate of 10 frames per second and the incident electron flux of <0.2 e^-1^ Å^–2^s^-1^.

Movie play speed: x 60 times of real speed

**Supporting movie 5**: In-situ transmission electron microscope imaging shows that the growth process of ZIF-67 cubic crystal with three nanoparticles attachment. It is a non-classical nucleation and growth mode.

Imaging conditions: The in-situ transmission electron microscope study was conducted in a 120kV TEM-1400 flash TEM (JEOL Co., Ltd., Tokyo, Japan) equipped with Gatan camera (Gatan Company, Pleasanton, California, USA). In-situ image series were obtained at the rate of 10 frames per second and the incident electron flux of <0.2 e^-1^ Å^–2^s^-1^.

Movie play speed: x 25 times of real speed

**Supporting movie 6**: In-situ transmission electron microscope imaging shows that the growth process of a single ZIF-67 cubic crystal, a classical nucleation and growth mode.

Imaging conditions: The in-situ transmission electron microscope study was conducted in a 120kV TEM-1400 flash TEM (JEOL Co., Ltd., Tokyo, Japan) equipped with Gatan camera (Gatan Company, Pleasanton, California, USA). In-situ image series were obtained at the rate of 10 frames per second and the incident electron flux of <0.2 e^-1^ Å^–2^s^-1^.

Movie play speed: x 60 times of real speed

**Supporting movie 7**: 3D visualization of ZIF-67 after UHS sintering at 1000°C for 1 minute.

Imaging condition: the TEM sample holder is rotated from -64˚ to +60˚, rotating by 2 degrees each time, and capturing a series of TEM images. The overall sampling time is approximately 30 minutes. These images are then used for 3D reconstruction. TEM images were collected via a 120 kV TEM-1400 Flash TEM (JEOL Ltd., Tokyo, Japan) equipped with a Gatan camera (Gatan Inc., Pleasanton, CA, USA).

**Supporting movie 8**: 3D visualization of ZIF-67 cross section after UHS sintering at 1000°C for 1 minute. (The same sample with Movie 7)

It is note that the reproducibility of the in-situ TEM imaging experiments is high (80%) based on our trials (4/5). The failure is caused by the broken of the graphene liquid pocket in the assembly process, not in the imaging process.

**Reference**

(1) Wang, W.; Yan, H.; Anand, U.; Mirsaidov, U. Visualizing the conversion of metal–organic framework nanoparticles into hollow layered double hydroxide nanocages. *Journal of the American Chemical Society* **2021**, *143* (4), 1854-1862.

(2) Chang, Q.; Yang, D.; Zhang, X.; Ou, Z.; Kim, J.; Liang, T.; Chen, J.; Cheng, S.; Cheng, L.; Ge, B. Understanding ZIF particle chemical etching dynamics and morphology manipulation: in situ liquid phase electron microscopy and 3D electron tomography application. *Nanoscale* **2023**, *15* (33), 13718-13727.

(3) Sun, Y.; Zhang, X.; Huang, R.; Yang, D.; Kim, J.; Chen, J.; Ang, E. H.; Li, M.; Li, L.; Song, X. Revealing microscopic dynamics: in situ liquid-phase TEM for live observations of soft materials and quantitative analysis via deep learning. *Nanoscale* **2024**, *16* (6), 2945-2954.

(4) Luo, B.; Smith, J. W.; Ou, Z.; Chen, Q. Quantifying the self-assembly behavior of anisotropic nanoparticles using liquid-phase transmission electron microscopy. *Accounts of chemical research* **2017**, *50* (5), 1125-1133.

(5) Tan, S. F.; Chee, S. W.; Lin, G.; Mirsaidov, U. Direct observation of interactions between nanoparticles and nanoparticle self-assembly in solution. *Accounts of chemical research* **2017**, *50* (6), 1303-1312.

(6) Loh, N. D.; Sen, S.; Bosman, M.; Tan, S. F.; Zhong, J.; Nijhuis, C. A.; Král, P.; Matsudaira, P.; Mirsaidov, U. Multistep nucleation of nanocrystals in aqueous solution. *Nature chemistry* **2017**, *9* (1), 77-82.

(7) Luo, B.; Wang, Z.; Curk, T.; Watson, G.; Liu, C.; Kim, A.; Ou, Z.; Luijten, E.; Chen, Q. Unravelling crystal growth of nanoparticles. *Nature nanotechnology* **2023**, *18* (6), 589-595.

(8) Smeets, P. J.; Finney, A. R.; Habraken, W. J.; Nudelman, F.; Friedrich, H.; Laven, J.; De Yoreo, J. J.; Rodger, P. M.; Sommerdijk, N. A. A classical view on nonclassical nucleation. *Proceedings of the National Academy of Sciences* **2017**, *114* (38), E7882-E7890.

(9) Banner, D. J.; Firlar, E.; Rehak, P.; Phakatkar, A. H.; Foroozan, T.; Osborn, J. K.; Sorokina, L. V.; Narayanan, S.; Tahseen, T.; Baggia, Y. In Situ Liquid‐Cell TEM Observation of Multiphase Classical and Nonclassical Nucleation of Calcium Oxalate. *Advanced Functional Materials* **2021**, *31* (18), 2007736.

(10) Wang, R.; Dong, Q.; Wang, C.; Hong, M.; Gao, J.; Xie, H.; Guo, M.; Ping, W.; Wang, X.; He, S. High‐temperature ultrafast sintering: exploiting a new kinetic region to fabricate porous solid‐state electrolyte scaffolds. *Advanced Materials* **2021**, *33* (34), 2100726.

(11) Xie, H.; Champagne III, V. K.; Zhong, W.; Clifford, B.; Liu, S.; Hu, L.; Zhao, J. C.; Clarke, D. R. Design, Fabrication, and Screening of Environmental‐Thermal Barrier Coatings Prepared by Ultrafast High‐Temperature Sintering. *Advanced Functional Materials* **2024**, *34* (10), 2309978.

(12) Wang, C.; Zhong, W.; Ping, W.; Lin, Z.; Wang, R.; Dai, J.; Guo, M.; Xiong, W.; Zhao, J. C.; Hu, L. Rapid synthesis and sintering of metals from powders. *Advanced Science* **2021**, *8* (12), 2004229.

(13) Lu, Q.; Wu, H.; Zheng, X.; Cao, Y.; Li, J.; Wang, Y.; Wang, H.; Zhi, C.; Deng, Y.; Han, X. Controllable constructing janus homologous heterostructures of transition metal alloys/sulfides for efficient oxygen electrocatalysis. *Advanced Energy Materials* **2022**, *12* (42), 2202215.

(14) Chen, L.; Huang, X.; Ma, R.; Xiang, W.; Ma, J.; Wu, Y.; Yang, D.; Wang, C.; Ping, W.; Xiang, H. A nanocrystal garnet skeleton-derived high-performance composite solid-state electrolyte membrane. *Energy Storage Materials* **2024**, *65*, 103140.

(15) Shi, J.; Li, R.; Zhang, J.; Wang, Y.; Ma, W.; Yue, Z.; Jin, C.; Liu, Y.; Zheng, L.; Bai, J. N-Coordinated Iridium–Molybdenum Dual-Atom Catalysts Enabling Efficient Bifunctional Hydrogen Electrocatalysis. *ACS Applied Materials & Interfaces* **2023**, *16* (1), 889-897.

(16) Kermani, M.; Dong, J.; Biesuz, M.; Linx, Y.; Deng, H.; Sglavo, V. M.; Reece, M. J.; Hu, C.; Grasso, S. Ultrafast high-temperature sintering (UHS) of fine grained α-Al2O3. *Journal of the European Ceramic Society* **2021**, *41* (13), 6626-6633.

(17) Podder, C.; Gong, X.; Pan, H. Ultrafast, Non‐Equilibrium and Transient Heating and Sintering of Nanocrystals for Nanoscale Metal Printing. *Small* **2021**, *17* (50), 2103436.

(18) Li, Z.; Wu, H. B.; Lou, X. W. D. Rational designs and engineering of hollow micro-/nanostructures as sulfur hosts for advanced lithium–sulfur batteries. *Energy & Environmental Science* **2016**, *9* (10), 3061-3070.

(19) Guo, C.; Liu, M.; Gao, G. K.; Tian, X.; Zhou, J.; Dong, L. Z.; Li, Q.; Chen, Y.; Li, S. L.; Lan, Y. Q. Anthraquinone covalent organic framework hollow tubes as binder microadditives in Li− S batteries. *Angewandte Chemie* **2022**, *134* (3), e202113315.

(20) Li, Q.; Song, Y.; Xu, R.; Zhang, L.; Gao, J.; Xia, Z.; Tian, Z.; Wei, N.; Rümmeli, M. H.; Zou, X. Biotemplating growth of nepenthes-like N-doped graphene as a bifunctional polysulfide scavenger for Li–S batteries. *ACS nano* **2018**, *12* (10), 10240-10250.

(21) Xing, Z.; Li, G.; Sy, S.; Chen, Z. Recessed deposition of TiN into N-doped carbon as a cathode host for superior Li-S batteries performance. *Nano Energy* **2018**, *54*, 1-9.

(22) Chen, X.; Zeng, S.; Muheiyati, H.; Zhai, Y.; Li, C.; Ding, X.; Wang, L.; Wang, D.; Xu, L.; He, Y. Double-shelled Ni–Fe–P/N-doped carbon nanobox derived from a prussian blue analogue as an electrode material for K-ion batteries and Li–S batteries. *ACS Energy Letters* **2019**, *4* (7), 1496-1504.

(23) Gai, L.; Zhao, C.; Zhang, Y.; Hu, Z.; Shen, Q. Constructing a multifunctional mesoporous composite of metallic cobalt nanoparticles and nitrogen‐doped reduced graphene oxides for high‐performance lithium–sulfur batteries. *Carbon Energy* **2022**, *4* (2), 142-154.

(24) Wang, Z.; Shen, J.; Ji, S.; Xu, X.; Zuo, S.; Liu, Z.; Zhang, D.; Hu, R.; Ouyang, L.; Liu, J. B, N codoped graphitic nanotubes loaded with Co nanoparticles as superior sulfur host for advanced Li–S batteries. *Small* **2020**, *16* (7), 1906634.

(25) Jin, L.; Chen, J.; Fu, Z.; Qian, X.; Cheng, J.; Hao, Q.; Zhang, K. ZIF-8/ZIF-67 derived ZnS@ Co-NC hollow core-shell composite and its application in lithium‑sulfur battery. *Sustainable Materials and Technologies* **2023**, *35*, e00571.

(26) Zhou, J.; Lin, N.; long Cai, W.; Guo, C.; Zhang, K.; Zhou, J.; Zhu, Y.; Qian, Y. Synthesis of S/CoS2 nanoparticles-embedded N-doped carbon polyhedrons from polyhedrons ZIF-67 and their properties in lithium-sulfur batteries. *Electrochimica Acta* **2016**, *218*, 243-251.

(27) Wang, J.; Chen, L.; Chen, X.; Li, X.; Xiao, J. ZIF-67 derived material encapsulated V2O3 hollow sphere structure of Co-NC@ V2O3-sp used as a positive electrode material for lithium-sulfur batteries. *Electrochimica Acta* **2024**, *474*, 143573.

(28) Liu, Z.; Wang, Q.; Wang, Z.; Ma, Y.; Wang, Y.; Du, Y.; Lei, W. Cherry blossom derived micron carbon sheets loaded with Co based particles served as sulfur host for lithium sulfur batteries. *Colloids and Surfaces A: Physicochemical and Engineering Aspects* **2024**, *685*, 133171.

(29) Wang, Y.; Zhang, R.; Chen, J.; Wu, H.; Lu, S.; Wang, K.; Li, H.; Harris, C. J.; Xi, K.; Kumar, R. V. Enhancing catalytic activity of titanium oxide in lithium–sulfur batteries by band engineering. *Advanced Energy Materials* **2019**, *9* (24), 1900953.

(30) Wu, R.; Chen, S.; Deng, J.; Huang, X.; Song, Y.; Gan, R.; Wan, X.; Wei, Z. Hierarchically porous nitrogen-doped carbon as cathode for lithium–sulfur batteries. *Journal of energy chemistry* **2018**, *27* (6), 1661-1667.

(31) Peng, H. J.; Huang, J. Q.; Zhao, M. Q.; Zhang, Q.; Cheng, X. B.; Liu, X. Y.; Qian, W. Z.; Wei, F. Nanoarchitectured graphene/CNT@ porous carbon with extraordinary electrical conductivity and interconnected micro/mesopores for lithium‐sulfur batteries. *Advanced functional materials* **2014**, *24* (19), 2772-2781.

(32) Ma, L.; Qian, J.; Li, Y.; Cheng, Y.; Wang, S.; Wang, Z.; Peng, C.; Wu, K.; Xu, J.; Manke, I. Binary Metal Single Atom Electrocatalysts with Synergistic Catalytic Activity toward High‐Rate and High Areal‐Capacity Lithium–Sulfur Batteries. *Advanced Functional Materials* **2022**, *32* (51), 2208666.

(33) Wu, Y.; Li, D.; Pan, J.; Sun, Y.; Huang, W.; Wu, M.; Zhang, B.; Pan, F.; Shi, K.; Liu, Q. Realizing fast polysulfides conversion within yolk-shelled NiO@ HCSs nanoreactor as cathode host for high-performance lithium-sulfur batteries. *Journal of Materials Chemistry A* **2022**, *10* (30), 16309-16318.

(34) Yang, D.; Han, Y.; Li, M.; Li, C.; Bi, W.; Gong, Q.; Zhang, J.; Zhang, J.; Zhou, Y.; Gao, H. Highly Conductive Quasi‐1D Hexagonal Chalcogenide Perovskite Sr8Ti7S21 with Efficient Polysulfide Regulation in Lithium‐Sulfur Batteries. *Advanced Functional Materials* **2024**, 2401577.
